# Sphingomyelin synthase inhibitors from edible mushrooms prevent overweight and improve vitamin D homeostasis in a mouse model of diet-induced obesity

**DOI:** 10.3389/fnut.2025.1658130

**Published:** 2025-12-10

**Authors:** Enkhmaa Enkhbat, Yuta Murai, Kohei Yuyama, Hui Sun, Mahadeva M. M. Swamy, Yoshiko Suga, Masaki Anetai, Kenji Monde

**Affiliations:** 1 Graduate School of Life Science, Hokkaido University, Sapporo, Japan; 2 Laboratory of Mammalian Ecology, Institute of Biology, Mongolian Academy of Sciences, Ulaanbaatar, Mongolia; 3 Faculty of Advanced Life Science, Hokkaido University, Sapporo, Japan

**Keywords:** sphingomyelin synthase, grifolin, grifolic acid, vitamin D, obesity

## Abstract

**Introduction:**

Sphingomyelin synthase (SMS) is a key enzyme in sphingolipid metabolism that converts phosphatidylcholine and ceramide into sphingomyelin and diacylglycerol, thereby influencing lipid signaling and metabolic regulation. Identifying natural SMS inhibitors is of interest for the prevention and management of obesity and related disorders.

**Methods:**

We screened more than 860 extracts from medicinal plants and mushrooms using a cell-based assay for SMS inhibition. Active fractions were purified, and grifolin and grifolic acid were isolated from the edible mushroom *Albatrellus confluens* (Alb. & Schwein.) Kotl. & Pouzar and structurally confirmed. Their inhibitory activity was validated *in vitro* against sphingomyelin synthase 1 (SMS1) and sphingomyelin synthase 2 (SMS2), and their metabolic effects were tested by dietary supplementation (0.1% in high-fat diet) in male C57BL/6J mice for 9 weeks.

**Results:**

Both compounds inhibited SMS activity at low micromolar concentrations. *In vivo*, they significantly reduced body weight gain, improved glucose tolerance, and alleviated hepatic steatosis in high-fat diet–fed mice. Treatment also preserved circulating 25-hydroxyvitamin D₃ [25(OH)D₃] concentrations and downregulated hepatic expression of the vitamin D–catabolizing enzyme CYP24A1.

**Conclusion:**

These findings demonstrate, for the first time, that mushroom-derived metabolites can act as dual-substrate SMS inhibitors with beneficial effects on lipid and vitamin D metabolism. Grifolin and grifolic acid thus represent promising candidates for development as functional food components or nutraceuticals to prevent obesity and metabolic syndrome.

## 1 Introduction

Overweight and obesity are major global public health challenges. According to the World Health Organization (2024), the prevalence of obesity has more than doubled since 1990. By 2022, approximately 43% of adults worldwide were classified as overweight and 16% as obese.^1^ Obesity and insulin resistance are key contributors to metabolic syndrome and increase the risk of cardiovascular disease, type 2 diabetes mellitus (T2DM), fatty liver disease, and certain cancers. Identifying effective strategies to prevent and control these conditions remains a critical public health priority.

Sphingolipid metabolism plays crucial roles in disease pathogenesis and has emerged as a promising therapeutic target. Among the major sphingolipids, ceramide (Cer), sphingomyelin (SM), and sphingosine-1-phosphate (S1P) function as bioactive signaling molecules regulating apoptosis, proliferation, and inflammation (1–3). The balance among these lipids is decisive: ceramide accumulation promotes apoptosis and metabolic dysfunction, whereas S1P and SM support survival and membrane stability. Disruption of this balance contributes to insulin resistance, hepatic steatosis, and chronic inflammation (4–12).

In mammals, sphingolipid metabolism is regulated by approximately 40 enzymes (13), including sphingomyelin synthase (SMS), which catalyzes the transfer of a phosphocholine group from phosphatidylcholine (PC) to ceramide, generating SM and diacylglycerol (DAG). SMS therefore regulates the Cer/SM ratio—a critical determinant of lipid homeostasis. Sphingomyelin synthase 1 (SMS1) and sphingomyelin synthase 2 (SMS2) are the major isoforms, localized to the Golgi and plasma membrane, respectively (14). Dysregulated SMS activity promotes lipid accumulation, insulin resistance, and obesity-related metabolic disorders (15). Notably, SMS2 deficiency in mice reduces adipose mass, alleviates fatty liver, and enhances glucose tolerance, partly through increased energy expenditure and the browning of white adipose tissue. These findings highlight SMS2 as a promising therapeutic target for obesity and metabolic syndrome (16, 17).

Small-molecule SMS inhibitors are therefore of high interest as potential therapeutic agents and functional food components. Natural products, in particular, offer structural diversity, biological compatibility, and favorable safety profile (18). However, despite their therapeutic promise, the precise enzymatic targets of natural compounds often remain poorly characterized (19). The therapeutic properties of mushrooms have been recognized for thousands of years in Eastern countries because they exhibit a wide range of biological activities. Mushrooms are especially attractive in this context: traditionally valued in Eastern medicine for their health-promoting properties, they represent a vast and chemically diverse resource, with ~140,000 estimated species but only ~10% characterized to date (20).

Building on our earlier identification of natural SMS inhibitors such as ginkgolic acid, malabaricones, and daurichromenic acid (21–23), we constructed a methanol-extract library of 212 mushroom species from Japan and screened them using a high-throughput, cell-based SMS assay. Extracts from *Albatrellus dispansus* (Lloyd) Pouzar and the edible *Albatrellus confluens* (Alb. & Schwein.) Kotl. & Pouzar exhibited the strongest activity, leading to the isolation of two phenolic compounds, grifolin and grifolic acid.

Both compounds inhibited SMS1 and SMS2 with the half maximal inhibitory concentration (IC₅₀) values ranging from 0.6 to 18.3 μM depending on the assay pathway, with grifolic acid showing consistently greater potency. In HepG2 cells, treatment with 20 μM grifolin or grifolic acid significantly reduced lipid droplet accumulation (by 43 and 48%) without detectable cytotoxicity. In diet-induced obese mice, 0.1% oral administration attenuated body weight gain, improved glucose tolerance, and decreased hepatic steatosis.

To our knowledge, this is the first report identifying grifolin and grifolic acid as natural SMS inhibitors derived from edible mushrooms. These findings reveal a new class of dual-substrate, food-derived SMS inhibitors with potential applications as functional food components or nutraceuticals for preventing obesity and related metabolic disorders.

## 2 Materials and methods

### 2.1 Materials

Fruiting bodies of *A. confluens* and *A. dispansus* were collected in Japan (Mie, Tottori, Okayama, and Hiroshima Prefectures, 2019) and authenticated by mycologists. Additional mushroom specimens (212 species) collected in Hokkaido were cleaned, authenticated, and deposited in the Natural Product Library of Hokkaido University. Samples were extracted with organic solvent, concentrated under reduced pressure, and stored at −20 °C. Crude extracts were dissolved in DMSO (10 mg/mL) for screening, resulting in a mushroom extract library of 212 species.

### 2.2 Cell culture and enzyme assays

Mouse embryonic fibroblasts lacking SMS1 and SMS2 (ZS2), and stable ZS2 lines expressing SMS1 or SMS2, were cultured in Dulbecco’s Modified Eagle medium (DMEM) supplemented with 10% fetal bovine serum at 37 °C in a 5% CO₂ incubator. Human hepatoma HepG2 cells were used for cytotoxicity and lipid uptake assays.

SMS activity was measured in cell lysates (0.1 μg/μL protein). Lysates were preincubated with test compounds at 37 °C for 30 min, followed by addition of C6-NBD-ceramide (NBD-Cer; N-[6-[(7-nitro-2-1,3-benzoxadiazol-4-yl)amino]hexanoyl]sphingosine, 5 μM) and C6-NBD-phosphatidylcholine (NBD-PC; 1-acyl-2-[6-[(7-nitro-2-1,3-benzoxadiazol-4-yl)amino]hexanoyl]-sn-glycero-3-phosphocholine, 5 μM) as substrates. The reaction converts ceramide (Cer) and phosphatidylcholine (PC) into sphingomyelin (SM) and diacylglycerol (DAG). Reactions were terminated with methanol/chloroform (1:2, v/v). The fluorescent products C6-NBD–diacylglycerol (C6-NBD-DAG) and C6-NBD–sphingomyelin (C6-NBD-SM) were separated and quantified by HPLC with fluorescence detection (λ_ex = 470 nm, λ_em = 530 nm). Assay linearity was verified with respect to both protein concentration and reaction time. Enzyme activity was calculated from product formation relative to control samples without inhibitor.

### 2.3 Cytotoxicity and lipid uptake assays

Cell viability was determined by the CCK-8 assay after 24 h incubation with 0–20 μM grifolin or grifolic acid. For lipid droplet accumulation experiments, HepG2 cells were treated with 1 mM oleic acid in the presence or absence of 20 μM compounds for 24 h. Cells were stained with Nile Red (λ_ex = 545 nm, λ_em = 605 nm) to visualize neutral lipids and with DAPI (λ_ex = 360 nm, λ_em = 460 nm) to label nuclei. Images were acquired using a Keyence BZ-X70 fluorescence microscope (Plan Apochromat 40x objective, fixed exposure times) and analyzed in ImageJ. Lipid droplet content was quantified by Otsu thresholding, expressed as Nile Red fluorescence intensity normalized to DAPI counts, yielding lipid content per cell in arbitrary units (AU/cell). Data are presented from three independent experiments, each including multiple fields of view, and statistical significance was assessed by one-way ANOVA with Tukey’s *post hoc* test. Fatty acid uptake assays such as BODIPY-C16 transport were not performed in this study, and our lipid accumulation measurements therefore reflect neutral lipid storage but not uptake kinetics.

### 2.4 Isolation and identification of active compounds

The acetone extract of *A. confluens* fruiting bodies (4.96 kg fresh weight) exhibited strong SMS inhibitory activity. Sequential liquid–liquid extraction with hexane, diethyl ether, and ethyl acetate was followed by silica gel chromatography. The ether fraction showed the greatest activity and yielded two phenolic compounds, grifolin (10.4 g) and grifolic acid (5.8 g). Structural identities were confirmed by NMR spectroscopy and high-resolution mass spectrometry, consistent with reported values.

### 2.5 Animal studies

All animal experiments were approved by the Animal Research Committee of Hokkaido University and performed in accordance with institutional guidelines. Male C57BL/6J mice (5 weeks old, Japan SLC Inc., Shizuoka, Japan) were housed under controlled environmental conditions (24 °C, 50% ± 10% humidity, 12 h light/dark cycle) with ad libitum access to food and water. After a one-week acclimatization period, animals were randomly assigned to four groups (*n* = 6 per group):

1. ND (normal diet; 6.2% kcal fat, AIN-93M, Oriental Yeast Co., Tokyo, Japan)
2. HFD (high-fat diet; 60% kcal fat, HFD-60, Oriental Yeast Co.)
3. HFD + grifolin (HFD supplemented with 0.1% grifolin)
4. HFD + grifolic acid (HFD supplemented with 0.1% grifolic acid).

Grifolin and grifolic acid were incorporated directly into the diet (AIN-93G, Oriental Yeast Co, Tokyo, Japan) and provided as oral dietary supplementation for 9 weeks. Body weight and food intake were monitored weekly.

Glucose tolerance was assessed after overnight fasting by oral gavage of glucose (1 g/kg body weight), and blood glucose was measured at 0, 30, 60, and 120 min. At study termination blood was collected by cardiac puncture under 2% isoflurane inhalation anesthesia.^2^ Liver and kidney tissues were excised, snap-frozen in liquid nitrogen, and stored at −80 °C for molecular analyses or processed for histological evaluation (hematoxylin and eosin, Oil Red O staining). Hepatic triglyceride content was measured enzymatically. Lipidomic profiling of ceramide and sphingomyelin species was conducted by Liquid chromatography–tandem mass spectrometry (LC–MS/MS). Serum 25(OH)D₃ levels were quantified using a commercial enzyme-linked immunosorbent assay (ELISA) kit, and hepatic and renal expression of CYP24A1, CYP27B1, and CYP2R1 was determined by quantitative reverse transcription PCR (RT–PCR).

Randomization was applied at group assignment, and investigators performing histological and biochemical analyses were blinded to treatment groups.

### 2.6 Statistical analysis

All experiments were performed at least in triplicate unless otherwise specified. Data are presented as mean ± standard deviation (SD). Statistical comparisons were performed using Student’s *t*-test or one-way ANOVA followed by Tukey’s *post hoc* test (GraphPad Prism 9). Differences were considered significant at *p* < 0.05.

## 3 Results

### 3.1 Screening and identification for SMS inhibitors

**Figure 1:**
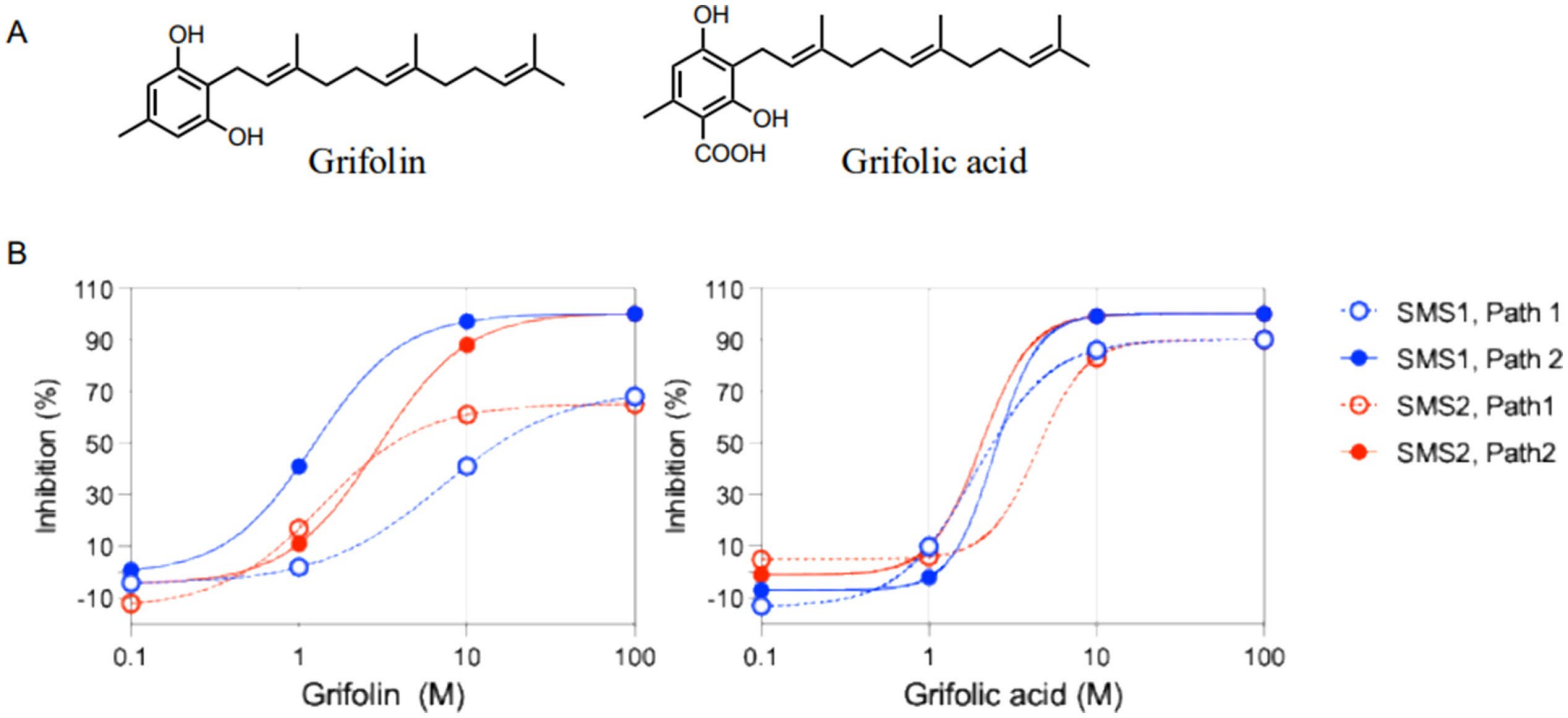
Grifolin and grifolic acid inhibit sphingomyelin synthase (SMS) activity *in vitro***. (A)** Chemical structures of grifolin and grifolic acid isolated from *A. confluens*. **(B)** Dose–dependent inhibition of SMS1 and SMS2. Enzyme assays were performed in ZS2 cells expressing SMS1 or SMS2 using 5 μM C6-NBD-ceramide (NBD-Cer) and 5 μM C6-NBD-phosphatidylcholine (NBD-PC) as substrates. Path 1 represents the conversion of NBD-PC to NBD-diacylglycerol (NBD-DAG), and Path 2 represents the conversion of NBD-Cer to NBD-sphingomyelin (NBD-SM). Products were quantified by HPLC with fluorescence detection. Linearity was verified with respect to protein concentration and incubation time. Data are mean ± SD (*n* ≥ 3).

To identify SMS inhibitors, we screened methanol and acetone extracts of the fruit bodies, leaves, stems, and roots of approximately 650 medicinal plants and 212 mushrooms from the Hokkaido University natural product library using a cell-based SMS assay (Supplementary Figures 1A,B). The full list of species screened is provided in Supplementary Table S2. We found that the fruiting body extracts of *A. dispansus* and *A. confluens* showed marked inhibitory activity against SMS (Supplementary Figures 1A,B). *A. dispansus* and *A. confluens* are edible and have been used as natural medicines, functional foods, and dietary supplements in China for thousands of years. Bioassay-guided fractionation of the *A. confluens* extract resulted in the identification of grifolin and grifolic acid as inhibitor compounds (Figure 1A). Each was confirmed by NMR spectroscopy, HRMS, and spectroscopic data, similar to previous reports (Supplementary Figure 1D). The dose-dependent inhibitory effects of grifolin and grifolic acid on SMS1 and SMS2 were validated using a cell-based assay, with both compounds exhibiting substantial inhibition across isoforms (Figure 1B).

Grifolin and grifolic acid were identified as major components of *Albatrellus* species. These compounds exhibited strong inhibitory activity against SMS using both PC and Cer substrates. SMS inhibitors were identified medicinal mushroom and plant extracts library of Hokkaido university, screening assays by using both fluorescence substrates, C6-NBD (N-[6-[(7-nitro-2-1,3-benzoxadiazol-4-yl)amino]hexanoyl])-Cer and C6-NBD-PC. The inhibitory activities of the extraction were quantified as follows: against the C6-NBD-PC substrate, both SMS1 and SMS2 showed inhibition at 4.5 μg/mL; against the C6-NBD-Cer substrate, SMS1 was inhibited at 0.5 μg/mL, and SMS2 at 0.3 μg/mL. The inhibitory effects of grifolin and grifolic acid are among the strongest (Supplementary Table 1) natural SMS inhibitors reported to date, targeting both substrates (21–23). The half maximal inhibitory concentration (IC₅₀) was defined as the compound concentration required to reduce enzyme activity by 50%. The estimated IC₅₀ values for grifolin were: 15.2 μM (SMS1, C6-NBD-PC), 18.3 μM (SMS2, C6-NBD-PC), 0.6 μM (SMS1, C6-NBD-Cer), and 3.0 μM (SMS2, C6-NBD-Cer). For grifolic acid, the IC₅₀ values were 3.0 μM (SMS1, C6-NBD-PC), 4.0 μM (SMS2, C6-NBD-PC), 3.0 μM (SMS1, C6-NBD-Cer), and 2.5 μM (SMS2, C6-NBD-Cer) (Table 1).

**Table 1:** IC₅₀ values of grifolin and grifolic acid against SMS1 and SMS2.

Compounds	IC_50_ (μM)			
	SMS1		SMS2	
	Path 1	Path 2	Path 1	Path 2
Grifolin	15.2	0.6	18.3	3
Grifolic acid	3	3	4	2.5

Assays used ZS2 cell lysates expressing SMS1 or SMS2 with 5 μM NBD-Cer and 5 μM NBD-PC. Path 1 = NBD-PC → NBD-DAG; Path 2 = NBD-Cer → NBD-SM. Values are from nonlinear regression (4-parameter logistic) of concentration–response curves; mean ± SD, *n* = 3 independent experiments. All units are μM.

### 3.2 Cell viability and inhibition of lipid droplet accumulation *in vitro*

**Figure 2:**
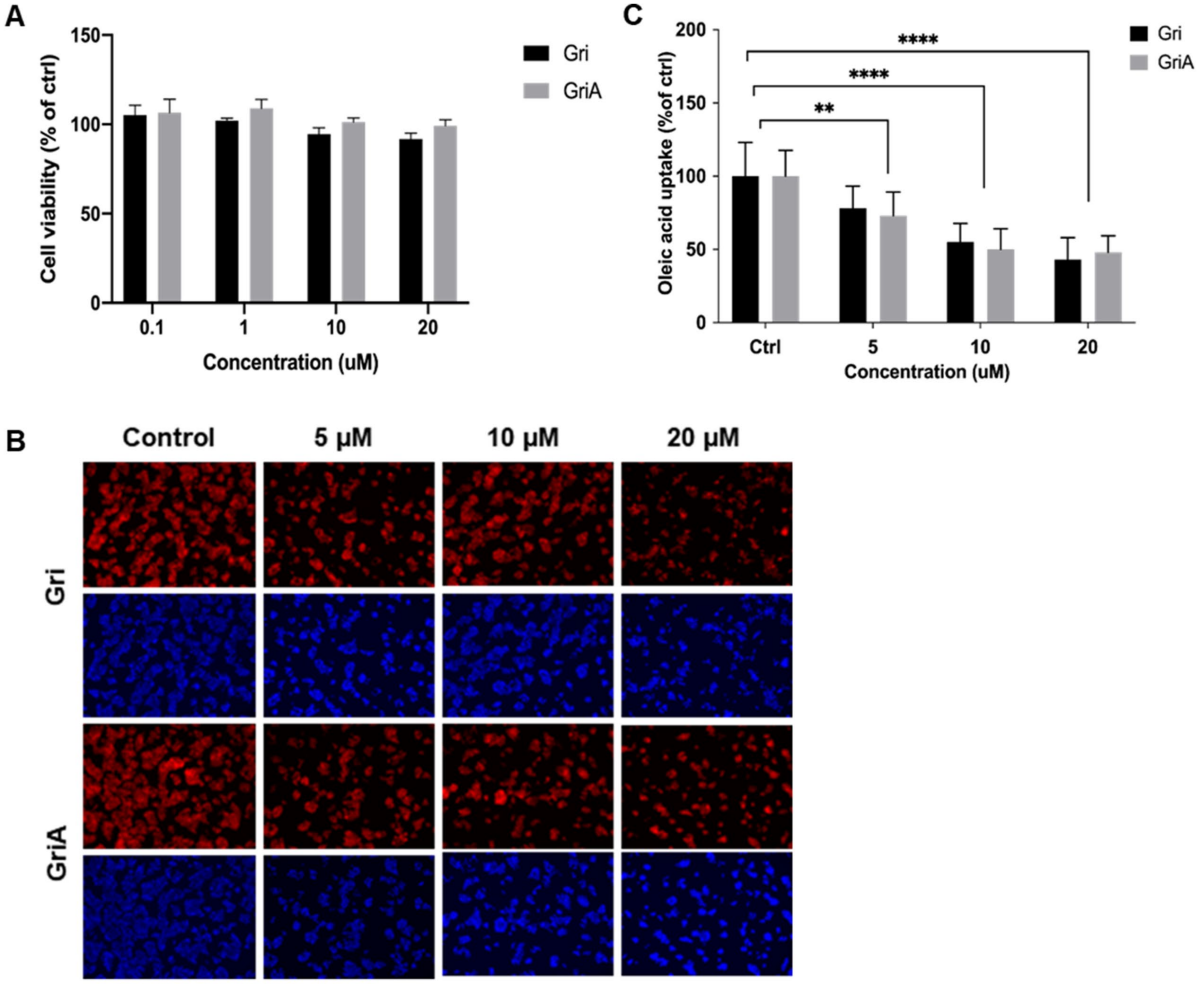
Effects of grifolin and grifolic acid on cell viability and lipid droplet accumulation in HepG2 cells. **(A)** Cell viability by CCK-8 after 24 h incubation with 0–20 μM grifolin or grifolic acid. **(B,C)** Nile Red (neutral lipids) and DAPI (nuclei) fluorescence images after 24 h exposure to 1 mM oleic acid (OA) in the absence or presence of 20 μM compounds. Imaging: Keyence BZ-X70, 20 × objective, fixed exposure across conditions; scale bar = 100 μm (applies to all panels). Quantification used ImageJ (Otsu thresholding); lipid content reported as Nile Red intensity per cell (AU/cell; normalized to DAPI counts). Data are mean ± SD, *n* = 3 independent experiments (multiple fields of view per replicate). Statistics: one-way ANOVA with Tukey’s *post hoc* test; significance indicated as: (*) *p* < 0.05, (**) *p* < 0.01, (***) *p* < 0.001, (****) *p* < 0.0005, ns = not significant.

We examined cytotoxicity in human hepatoma HepG2 cells. Neither compound showed significant cytotoxicity up to 20 μM (Figure 2A). SMS-deficient cells and mice have previously been reported to exhibit impaired fatty acid uptake and lipid droplet formation (16, 24). To investigate whether the identified SMS inhibitors affected lipid accumulation, we assessed lipid droplet formation in HepG2 cells following treatment with oleic acid (OA). Lipid droplets, which were stained with Nile Red, were remarkably reduced by both grifolin and grifolic acid treatment in a dose-dependent manner (grifolin treated 43% and grifolic acid treated 48% of lipid reduction at 20 μM, Figures 2B,C). Building on a previous report that oral administration of grifolin at 2,000 mg/kg was safe in mammals (25), we conducted an *in vivo* study using a high-fat diet-induced obesity mouse model.

### 3.3 Grifolin and grifolic acid prevent HFD-induced obesity

**Figure 3:**
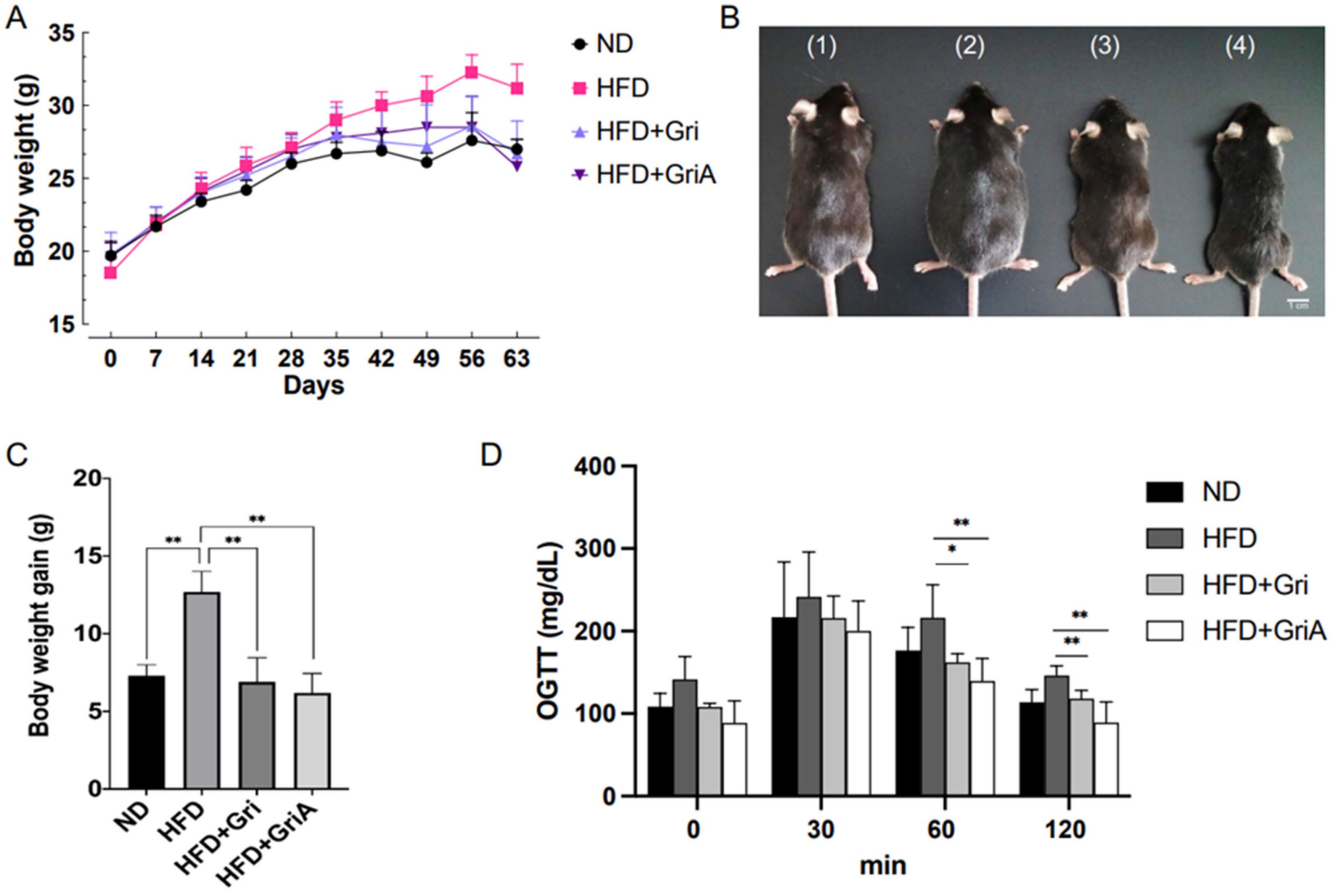
*In vivo* effects of grifolin and grifolic acid on body weight and glucose tolerance in diet-induced obese mice. **(A)** Weekly body weight gain; **(B)** Representative images of mice: (1) ND, (2) HFD, (3) HFD + 0.1% grifolin, (4) HFD + 0.1% grifolic acid; **(C)** Final body weight after 9 weeks; **(D)** Blood glucose levels during oral glucose tolerance test (OGTT; 1 g/kg, *n* = 6 per group) at 0, 30, 60, and 120 min; Statistical analysis one-way ANOVA with Tukey’s post hoc test: (*) *p* < 0.05, (**) *p* < 0.01, (***) *p* < 0.001, (****) *p* < 0.0005. Feed intake data are provided in Supplementary Figure 2.

Oral dietary supplementation with grifolin or grifolic acid (0.1% in HFD, corresponding to ~100 mg/kg/day) for 9 weeks markedly reduced body weight gain in male C57BL/6J mice (*n* = 6 per group). The mean final body weights were: ND – 27.0 g, HFD – 31.2 g, HFD + grifolin – 26.7 g, and HFD + grifolic acid – 25.8 g. When compared to the HFD group, this corresponds to a relative reduction of 14.6% for grifolin and 17.4% for grifolic acid (Figures 3A,C; representative images in Figure 3B).

Feed intake was recorded weekly and differed significantly among groups (Supplementary Figure 2). HFD-fed mice consumed significantly less food (3.02 ± 0.54 g/day) compared with HFD + Gri (4.68 ± 1.24 g/day, *p* < 0.05) and HFD + GA (4.82 ± 1.06 g/day, *p* < 0.05), while intake in the treated groups did not differ significantly from ND controls (4.55 ± 0.8 g/day). Thus, the reduced body weight observed in the treated groups was not attributable to reduced caloric intake but rather to the metabolic effects of grifolin and grifolic acid.

We investigated the effects of these inhibitors on glucose tolerance, hepatic TG levels, and lipid droplet accumulation. As expected, after 9 weeks an oral glucose tolerance test (1 g/kg glucose by gavage following overnight fasting) showed that both grifolin- and grifolic acid–treated mice exhibited significantly improved glucose tolerance compared to HFD-group (Figure 3D).

### 3.4 Grifolin and grifolic acid prevent liver steatosis

**Figure 4:**
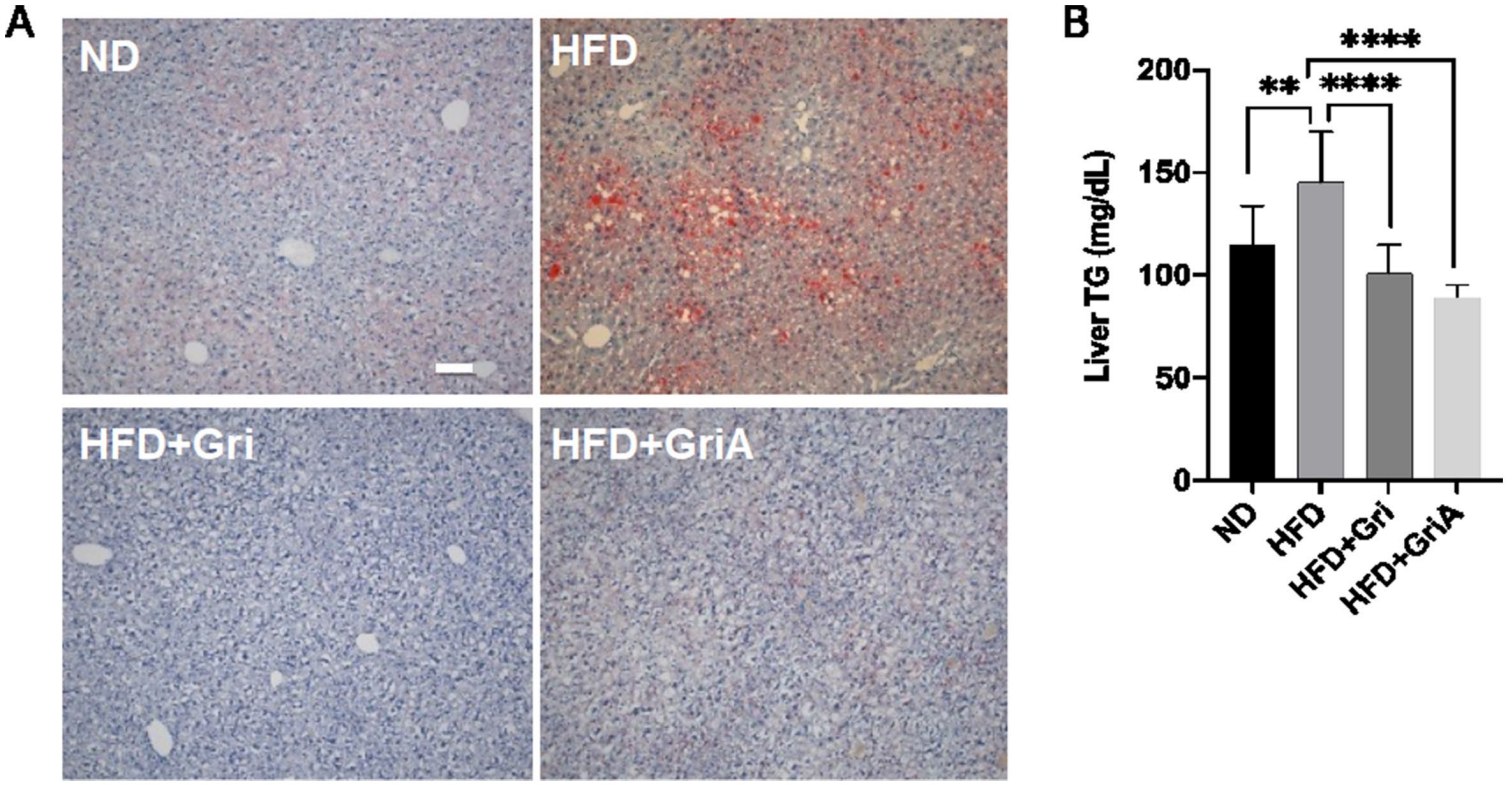
Effects of grifolin and grifolic acid on hepatic lipid accumulation in diet-induced obese mice. **(A)** Representative Oil Red O–stained liver sections from ND, HFD, HFD + Gri, and HFD + GA groups (*n* = 3 mice per group, independent samples). Scale bar = 100 μm (applies equally to all panels). **(B)** Hepatic triglyceride levels (mg/dL) in ND, HFD, HFD + Gri, and HFD + GA groups (*n* = 6 mice per group). *T*-test: (*) *p* < 0.05, (**) *p* < 0.01, (***) *p* < 0.001, (****) *p* < 0.0005, ns = not significant versus control.

In agreement with the results obtained in HepG2 cells, the histological study of the Oil Red O staining demonstrated that the treatment with grifolin and grifolic acid prevented lipid accumulation in the liver of HFD-fed mice (Figure 4A). Conversely, the untreated HFD-fed mice showed substantial lipid droplet accumulation, indicative of liver steatosis. In contrast, the treated groups showed improved lipid metabolism and minimal lipid deposition in the liver (Figure 4A). Hepatic triglyceride (TG levels in the HFD group were 30.5 and 38.4% higher than those in the grifolin-treated and grifolic acid-treated groups, respectively Figure 4B).

Subsequently, we measured SM levels in liver tissues collected from control and grifolin-treated HFD mice. As expected, in the liver tissues of grifolin-treated mice, SM levels were significantly reduced, suggesting suppressed SMS activity (Supplementary Figure 3A). Consistent with the SM measurement results, analysis of SMS activity in liver tissues from control and grifolin-treated HFD mice showed that DAG and SM synthesized from PC and Cer were significantly reduced in the grifolin-treated group (Supplementary Figures 3B,C). Grifolic acid treatment also showed a trend toward reduced DAG and SM levels, although the differences were not statistically significant. Taken together, these results demonstrate that grifolin and grifolic acid suppress weight gain, prevent hepatic lipid droplet formation, and improve glucose tolerance, likely by suppressing SMS activity in HFD-induced obese mice.

### 3.5 Stabilization of vitamin D level in the serum by grifolin and grifolic acid

**Figure 5:**
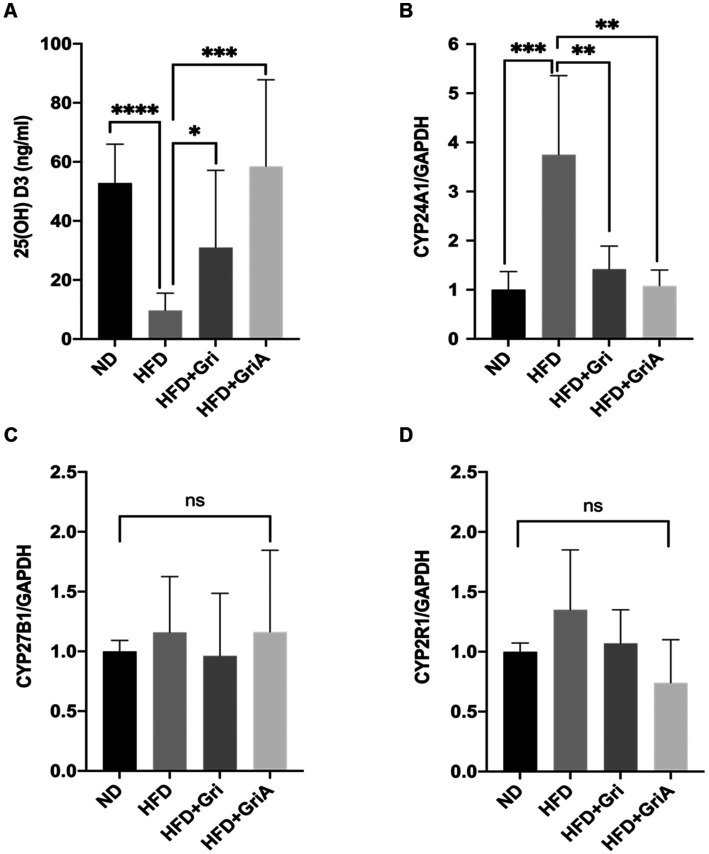
Effects of grifolin and grifolic acid on serum vitamin D levels and vitamin D–related gene expression in diet-induced obese mice. **(A)** Serum 25(OH)D₃ concentrations. **(B)** Renal CYP24A1 expression. **(C)** Hepatic CYP27B1 expression. **(D)** Hepatic CYP2R1 expression Groups: ND, HFD, HFD + Gri, HFD + GriA (*n* = 6 mice per group). *T*-test: (*) *p* < 0.05, (**) *p* < 0.01, (***) *p* < 0.001, (****) *p* < 0.0005, ns = not significant.

We selected three genes based on a previous study and performed qRT-PCR on mRNA extracted from liver and kidney tissues of DIO mice after 9 weeks of treatment. The selected CYP24A1, CYP27B1, and CYP2R1 genes showed involvement in 25(OH)D_3_ regulation. CYP27B1 is involved in the synthesis of active vitamin D, whereas CYP24A1 and CYP2R1 are involved in its catabolism. Serum 25(OH)D_3_ concentration significantly decreased in the HFD-fed group. In contrast, 25(OH)D_3_ levels were approximately 2.5-fold higher in the grifolin-fed group (*p* < 0.05) and approximately 6.3-fold higher in the grifolic acid-fed group (*p* < 0.001) than in the HFD-fed group (Figure 5A). The mRNA expression of CYP24A1 was significantly upregulated in the HFD-fed group (Figure 5B). Conversely, no significant differences in the CYP27B1 and CYP2R1 expression levels were observed between the groups (Figures 5C,D). These findings suggest that SMS inhibition by grifolin and grifolic acid may downregulate CYP24A1 expression, thereby reducing the active vitamin D catabolism and stabilizing the physiological levels of active 25(OH)D_3_.

## 4 Discussion

The exploration of enzyme inhibitors is a key strategy for developing low-molecular-weight drugs and biologically active compounds, substantially advancing the pharmaceutical field (19). In the present study, we identified two SMS inhibitors from natural sources: grifolin and grifolic acid. Consistent with the findings from studies using SMS-KO mice (16), these compounds were effective in reducing obesity, preventing hepatic steatosis, and improving glucose tolerance in a DIO model. In addition, treatment with grifolin or grifolic acid prevented the decrease in serum 25(OH)D_3_ levels typically observed in HFD-fed mice. These findings highlight SMS inhibitors as promising candidates for drugs or functional food development targeting obesity and enhance our understanding of the crosstalk between sphingolipid homeostasis and metabolic regulation.

Interestingly, although HFD-fed mice consumed less food than animals receiving grifolin or grifolic acid supplementation, they nevertheless gained more weight and exhibited more severe metabolic impairments. This indicates that the observed benefits cannot be explained by caloric intake alone but instead reflect modulation of lipid metabolism. This conclusion aligns with a growing body of evidence suggesting that obesity is not driven exclusively by energy intake, but also by alterations in lipid signaling pathways and adipose tissue dynamics (26, 27).

To identify SMS inhibitors, we screened a natural product extract library constructed from 650 plants and 212 mushrooms, either grown wild or cultivated in Hokkaido. This library included a local medicinal plant collection and was evaluated using a high-throughput cell-based SMS assay. The most potent inhibitory activity was observed in the preliminary screening of the ether extract from the fruiting bodies of the *A. confluens*. Mushrooms have been used in traditional Chinese medicine since ancient times. Their secondary metabolites such as polysaccharides (mainly *β*-D-glucans), heteroglycans, chitinous substances, peptidoglycans, proteoglycans, lectins, RNA components, lectins, lactones, alkaloids, terpenes, flavonoids, terpenoids, steroids, phenols, glycoproteins, nucleotides, fatty acids, vitamins, proteins, amino acids, antibiotics, and minerals, have been reported to exert various beneficial impacts on human health and provide protection against disease (28). Mushrooms have been shown to exert anti-obesity effects. Both long-term (1 year) and short-term (4-day) clinical trials involving obese or diabetic participants were conducted to evaluate the effects of substituting 20% high-energy beef with 20% low-energy white button mushrooms in the diet. These studies demonstrated that mushroom consumers exhibited lower BMI, decreased belly circumference, and increased satiety without compromising palatability (29, 30). Preliminary biological activities of *Albatrellus* species have been reported, including antioxidant (31), antifungal (32), and nitric oxide inhibitory (33) activities; neuroprotective effects in acute cerebral ischemia (25); and inhibition of lipopolysaccharide-induced B-lymphocyte proliferation (34). Chemical studies of *Albatrellus* have identified grifolin and grifolic acid as the major components, along with grifolic acid methyl ester, 3-hydroxyneogrifolin, 1-formylneogrifolin, 1-formyl-3-hydroxyneogrifolin, and a newly identified compound, grifolinol. Their chemical structures were determined using a combination of two-dimensional (2-D) NMR, mass spectroscopy, and chemical reactions (35).

Grifolin and grifolic acid are amphipathic compounds composed of a hydrophilic head group from salicylic acid and a common hydrophobic tail group from the hydrocarbon chain and have physical properties similar to those of sphingosine (Figure 1). Based on their structure, we hypothesized that grifolin and grifolic acid could inhibit SMS by acting as sphingolipid mimics. In this study, we identified for the first time that the SMS inhibitors grifolin and grifolic acids suppress SM and DAG synthesis. Both compounds significantly inhibited the DAG and SM formation *in vitro* (Table 1). We further confirmed that grifolin significantly reduced SM and DAG levels in the liver tissues of treated mice (Supplementary Figure 3). This dual inhibition is noteworthy because DAG synthesized by SMS2 has been reported to act as a second messenger that activates the PKC-JNK signaling pathway, thereby impairing insulin signaling (36). The potency of these compounds can also be placed in the context of other natural SMS inhibitors. As summarized in Supplementary Table 1, their IC₅₀ values are comparable to or stronger than previously reported inhibitors, such as ginkgolic acid, malabaricones, and daurichromenic acids (21–23). Grifolic acid, in particular, displayed submicromolar potency against SMS2 in the C6-NBD-ceramide assay, highlighting its potential as one of the most active natural SMS inhibitors identified to date.

A particularly novel observation is the effect of SMS inhibition on vitamin D metabolism. Both grifolin and grifolic acid preserved serum 25(OH)D₃ levels and downregulated renal expression of CYP24A1, the enzyme responsible for degrading 1,25(OH)₂D₃ (37). This is notable because vitamin D bioavailability is reduced in obesity due to sequestration in adipose tissue (38), and sphingolipid imbalances have been linked to vitamin D deficiency (39–42). Previous studies also suggest that vitamin D₃ can stimulate SMase activity (42) and that ceramide and SM levels are associated with dyslipidemia and vitamin D deficiency (39). Taken together, these findings suggest a novel cross-talk between sphingolipid metabolism and vitamin D pathways. Since both systems influence calcium homeostasis, inflammatory signaling, and insulin sensitivity, this interaction may represent an additional mechanism by which SMS inhibition improves metabolic outcomes.

Certain limitations should be acknowledged. Because substrate-varying kinetic analyses were not performed, the precise inhibition mechanism (competitive, mixed, or noncompetitive) with respect to ceramide or phosphatidylcholine remains undefined. Moreover, fatty acid uptake assays (e.g., BODIPY-C16 transport) were not conducted, leaving open the possibility that effects on fatty acid transport contribute to the observed outcomes. Cytotoxicity was assessed only using the CCK-8 assay, without further evaluation of membrane integrity or mitochondrial function. Additionally, lean body mass was not measured, limiting conclusions regarding body composition changes. Addressing these limitations through future studies—particularly kinetic modeling, fatty acid uptake analyses, extended cytotoxicity profiling, and body composition assessment—will provide a clearer mechanistic understanding. Finally, translation to human application will require careful investigation of pharmacokinetics, safety, and effective delivery strategies.

In conclusion, this study demonstrates that grifolin and grifolic acid act as dual-substrate SMS inhibitors with beneficial effects on obesity, glucose tolerance, hepatic lipid accumulation, and vitamin D metabolism in a DIO mouse model. These findings highlight the potential of mushroom-derived natural products as functional food ingredients or nutraceuticals for the prevention and management of obesity and related metabolic disorders. By targeting both lipid signaling and vitamin D pathways, grifolin and grifolic acid represent a unique class of multifunctional bioactives that warrant further investigation in translational and clinical settings.

## Data Availability

The datasets presented in this study can be found in online repositories. The names of the repository/repositories and accession number(s) can be found in the article/Supplementary material.

## Footnotes

1 World Health Organization. (2025). Obesity and overweight. Available from: https://www.who.int/news-room/fact-sheets/detail/obesity-and-overweight. [Accessed July 24, 2025].

2 American Veterinary Medical Association. Available from: https://www.avma.org/resources-tools/avma-policies/avma-guidelines-euthanasia-animals
