# Supplementary material for: Sphingomyelin synthase inhibitors from edible mushrooms prevent overweight and improve vitamin D homeostasis in a mouse model of diet-induced obesity
Source: Front Nutr. 2025 Dec 10;12:1658130. doi: 10.3389/fnut.2025.1658130 (PMC12728570; doi:10.3389/fnut.2025.1658130)
Supplement: Supplementary file 1 [file Data_Sheet_1.pdf]

Supplementary Material

Sphingomyelin synthase inhibitors from edible mushrooms prevent overweight and improve vitamin D homeostasis in a mouse model of diet-induced obesity

Enkhmaa Enkhbat[1,2], Yuta Murai[1,3], Kohei Yuyama[1,3], Hui Sun[3], Mahadeva M. M. Swamy[1,3], Yoshiko Suga[3], Masaki Anetai[3] and Kenji Monde[1,3*]

1. Graduate School of Life Science, Hokkaido University, Sapporo 001-0021, Japan.

2. Laboratory of Mammalian Ecology, Institute of Biology, Mongolian Academy of Sciences, Ulaanbaatar, 210351, Mongolia

3. Faculty of Advanced Life Science, Hokkaido University, Sapporo 001-0021, Japan.

* Corresponding Author: kmonde@sci.hokudai.ac.jp

Contents

・**Materials and methods**

・**SI Figure 1-3**

・**SI Table 1, 2**

・**References**

MATERIALS AND METHODS

Materials

A. confluens was collected from the west side of Japan, Mie, Tottori, Okayama and Hiroshima Prefectures, in October 2019. Mr. Eiji Nagasawa of Tottori Mycological Institute arranged the collection of specimens of the species. The specimens were identified by five collectors and sent to the Natural Product Library of Hokkaido University via refrigerated delivery and stored in a refrigerator until they were analyzed.

Preparation of mushroom extract library

From 2003 to 2005, a total of 212 mushroom specimens were collected in Hokkaido, Japan, and authenticated by Mr. Sadao Eisaka, Chairman of the Hokkaido Mushroom Advisory Association. Voucher specimens were deposited in the Natural Product Library of Hokkaido University. Collected fruiting bodies were carefully cleaned of soil and debris, frozen, and stored for subsequent research and analysis.

Fresh mushrooms typically contain approximately 90% water; thus, a 50 g fresh sample contains ~45 g (45 mL) of water. For extraction, 50 g of fresh material was treated with 105 mL of acetone, yielding a 150 mL solution of 70% aqueous acetone. Roughly chopped mushrooms were soaked overnight in acetone, filtered through a funnel, and the solvent was removed completely under reduced pressure using a rotary evaporator (N-1110, EYELA, Tokyo Rikakikai Co., Ltd., Tokyo, Japan). The resulting crude extracts were stored at −20 °C.

For screening, each dried extract was dissolved in dimethyl sulfoxide (DMSO; FUJIFILM Wako Pure Chemical Corp., Osaka, Japan) to a final concentration of 10 mg/mL, yielding a library of mushroom extracts suitable for sphingomyelin synthase (SMS) inhibitory assays.

Cell cultures

Wild-type mouse embryonic fibroblasts (MEF) and the reconstituted MEF lines ZS2/SMS1 and ZS2/SMS2 (6) were used in this study. ZS2 cells were derived from an SMS1/SMS2 double-KO MEF line, which was immortalized using the SV40 large T antigen. ZS2 cells stably expressing SMS1 or SMS2 were designated as ZS2/SMS1 and ZS2/SMS2, respectively. Human hepatoma HepG2 cells were obtained from the American Type Culture Collection. All cell lines were cultured at 37 ºC in

Dulbecco's Modified Eagle medium supplemented with 10% fetal bovine serum (FBS) in a humidified incubator with 5% CO_2.

Measurement of SMS inhibitor activity in cell lysate

Cell lysates were prepared as follows: ZS2/SMS1 and ZS2/SMS2 cells (protein concentration: 0.1 µg/µL) were resuspended in 20 mM Tris buffer (pH 7.5) and sonicated. Aliquots (100 µL) of cell lysates were incubated with 1 µL of the indicated inhibitor concentrations at 37°C for 30 minutes. After incubation, 1 µL of C6-NBD-ceramide (5 µM) and 1 µL of C6-NBD-phosphatidylcholine (Avanti Polar Lipids, Inc., Japan) were added to the mixture, and the reaction was further incubated at 37°C for 3 hours.

The reaction was terminated by adding 400 µL of methanol/chloroform [1/2 (v/v)], and the mixture was shaken and centrifuged at 1500 rpm for 5 minutes. C6-NBD-diacylglycerol and C6-NBD-sphingomyelin concentrations were quantified using high-performance liquid chromatography (HPLC). A reverse-phase HPLC assay was developed using a JASCO HPLC system, equipped with a PU-2089 Plus pump and FP-2020 Plus fluorescence detector, with excitation set to λex = 470 nm and emission at λem = 530 nm. A 50 × 4.6 mm YMC-Pack Diol-120-NP column (5 µm particle size) was used for separation, with a mobile phase consisting of isopropanol, hexane, and water, at a flow rate of 1.0 mL/min.

Screening of natural product library

To identify SMS inhibitors, we conducted a high-throughput screening of methanol extracts from a mushroom library using a previously described cell-based SMS assay. The entire mushroom library was extracted with 70% acetone, and the medicinal plant library was extracted with 80% methanol. Several extracts exhibited inhibitory activities against SMS (SI Figure 1C). Fruiting body extracts of *A. dispansus* and *A. confluens* displayed the most potent SMS inhibition. The fruiting body of *A. confluens* (4959 g) collected from Japan was fresh and thoroughly ground. After screening the active fractions, each residue was subjected to the SMS assay. The active components responsible for SMS inhibition were identified as grifolin and grifolic acid, confirmed by nuclear magnetic resonance (NMR) spectroscopy and high-resolution mass spectrometry (HRMS). The yields were as follows: grifolin (10.4 g) and grifolic acid (5.8 g).

Isolation of active compounds

Fruiting bodies of *Albatrellus confluens* (4,959 g, collected in Japan) were ground while fresh and extracted three times with 70% acetone (15.0 L) at room temperature, each extraction lasting 72 h. The combined acetone extracts were concentrated under reduced pressure to yield a dark brown oily residue (650 g). This residue was dissolved in 20% methanol in water (500 mL) and sequentially partitioned with hexane (3 × 250 mL), diethyl ether (Et_2O; 3 × 250 mL), and ethyl acetate (EtOAc; 3 × 250 mL). After solvent removal, each fraction was tested for SMS inhibitory activity. The Et_2O fraction (156 g) exhibited higher activity than the hexane fraction, while the aqueous fraction was inactive.

The active ether fraction was further purified by silica gel column chromatography. The major active components were identified as grifolin (10.4 g) and grifolic acid (5.8 g). Their structures were confirmed by NMR spectroscopy and HRMS, and the spectral data were consistent with previous reports in the literature. Grifolin and grifolic acid are among the major metabolites of *Albatrellus* species.

Grifolin was obtained as an orange-brown oil; HRMS (*m/z*): $[M - H]^-$, calculated for $C_{22}H_{31}O_2$: 327.2402; found: 327.2840

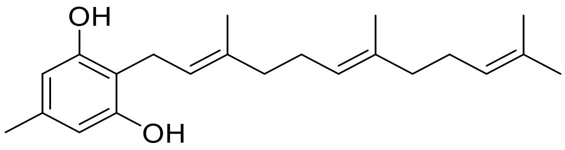

^{1}H NMR ($CDCl_3$, 500MHz): δ 6.25 (s, 2H), 5.29 (t, *J* = 7.1 Hz, 1H), 5.12–5.06 (m, 2H), 4.99 (s, 2H), 3.39 (d, *J* = 7.1 Hz, 2H), 2.22 (s, 3H), 2.15–1.94 (comp. m, 8H), 1.82 (s, 3H), 1.68 (s, 3H), 1.60 (comp. m, 6H)

^{13}C NMR (125 MHz, $CDCl_3$): δ = 154.8, 138.7, 137.5, 135.8, 135.6, 131.3, 124.4, 124.3, 123.6, 122.4, 121.6, 110.4, 109.1, 109.0, 108.7, 40.0, 39.7, 39.6, 36.9, 36.2, 32.7, 32.0, 26.7, 26.6, 26.3, 26.2, 25.7, 25.4, 23.3, 22.2, 21.9, 21.0, 20.5, 19.5, 17.7, 16.2, 16.0

Grifolic acid was obtained as a pale yellow, oily crystal; HRMS (*m/z*): $[M - H]^-$, calculated for $C_{23}H_{31}O_4$: 371.2301; found: 371.3003.

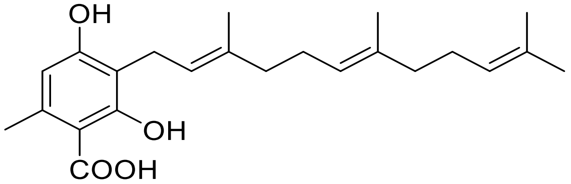

^{1}H NMR ($CDCl_3$, 500MHz): δ 11.87 (br. s, 1H), 6.27 (s, 1H), 5.29 (t, J = 7.0 Hz, 1H), 5.12–5.06 (m, 2H), 3.44 (d, J = 7.3 Hz, 2H), 2.54 (s, 3H), 2.19–1.96 (comp. m, 8H), 1.83 (s, 3H), 1.68 (s, 3H), 1.60 (comp. m, 6H)

13C NMR (125 MHz, $CDCl_3$): δ = 176.6, 164.0, 163.5, 160.4, 142.5, 141.9, 139.1, 135.8, 135.6, 131.6, 131.3, 124.9, 124.5, 124.4, 124.3, 123.6, 122.0, 121.3, 114.0, 111.8, 111.7, 111.6, 111.4, 111.2, 104.0, 40.0, 39.7, 39.6, 36.8, 35.7, 32.6, 32.0, 29.7, 26.7, 26.6, 26.3, 26.1, 25.7, 25.4, 24.2, 23.5, 23.4, 23.3, 22.0, 21.8, 20.3, 19.5, 17.7, 17.6, 16.2, 16.2, 16.0, 15.9.

Cell viability assay

Cell cytotoxicity was assessed using a Cell Counting Kit-8 (CCK-8; Dojindo Laboratories, Kumamoto, Japan) according to the manufacturer's protocol. HepG2 cells (2×10^5 cells/mL) were seeded in 96-well plates and incubated overnight. The cells were then treated with varying grifolin and grifolic acid concentrations (0, 0.01, 0.1, 1, 10, and 20 µM) and incubated at 37°C for 24 h. Following treatment, 10 µL of CCK-8 solution was added to each well, and the cells were incubated for an additional 1 hour at 37°C. The absorbance was measured at 450 nm using a microplate reader (iMark, Bio-Rad). Cell viability was calculated as a percentage of the absorbance of untreated control cells.

Uptake assay for oleic acid

HepG2 cells (1×10^5 cells/well) were seeded in a 12-well plate and incubated at 37 °C in 5% CO_2. The following day, the cells were treated with 1 mM of oleic acid (OA), OA plus 20 µM grifolin, or OA plus 20 µM grifolic acid in FBS-free medium. Cells were incubated for 24 h at 37 °C. After incubation, the cells were fixed with 4% paraformaldehyde and stained with 2.5 mg/mL of Nile Red solution for 15 min. After counterstaining with DAPI, images were captured using a BZ-X70 fluorescence microscope and analyzed with ImageJ software. OA uptake was expressed as a percentage of that in the control (untreated) cells.

Animals and diets

All animal procedures complied with the ethical guidelines of the Animal Research Committee of Hokkaido University. Male C57BL/6J mice (5-week-old; Japan SLC Inc., Shizuoka, Japan) were housed in a temperature- and humidity-controlled environment (24°C; 50% ± 10% relative humidity) with a 12-hour light/dark cycle. Mice were randomly divided into four experimental groups (n = 6 per group) as follows:

Normal diet: vehicle control group (6.2% calories from fat; AIN-93M; Oriental Yeast Co., Ltd., Tokyo, Japan)

High-fat diet (HFD): vehicle control group (60% calories from fat; HFD-60, Oriental Yeast Co., Ltd.)

HFD + 0.1% grifolin diet: test group

HFD + 0.1% grifolic acid diet: test group

Mice had ad libitum access to food and water. Body weight and food intake were recorded weekly throughout the 9-week experimental period. Blood samples were collected via intracardiac puncture. Liver tissues were carefully dissected, snap-frozen in liquid nitrogen, and stored at −80°C until further analysis.

Glucose tolerance test

The glucose tolerance test (GTT) was performed by oral administration of a glucose solution (1 g/kg of body weight) after overnight fasting. Blood glucose levels were measured using an ACCU-CHEK Aviva glucometer (Roche, Switzerland) at 0 min (baseline) and 30, 60, and 120 min after the oral glucose load.

Tissue histology

Liver and kidney samples from each group were reselected and fixed in 4% paraformaldehyde in phosphate buffer with saline PBS (pH 7.4). Tissue sections were stained with Oil Red O and hematoxylin and eosin. Images were captured using the Axio Imager M2 (Carl Zeiss).

Measurement of cellular and hepatic triglyceride (TG)

TG levels were measured as previously described (1,2) using a commercial TG assay kit (Wako, Osaka, Japan) following the manufacturer's instructions. HepG2 cells (1×10^5/well) were seeded in 12-well plates and treated with 1 mM oleic acid in serum-free medium for 24 h. After incubation, TG content was determined by enzymatic hydrolysis and colorimetric detection with DAOS [N-ethyl-. N-(2-

hydoxy-3-sulfopropyl)-3,5-dimethoxyaniline] chromogen at 550 nm using a microplate reader. Hepatic TG content was measured from ~50 mg of frozen liver tissue homogenized in 1 mL extraction buffer. TG levels were expressed as mg/g liver tissue or normalized to protein content for cells.

Measurement of SMS activity in liver tissues

Liver samples were weighted, homogenized, and centrifuged to get supernatant as lysates solution. The reaction and analysis were similar as previously described in cell lysate assays.

Measurement of 25(OH)D_3

Serum 25(OH)D_3 levels were measured using a 25(OH) Vitamin D Elisa kit (Wako, Japan), following the manufacturer's instructions. The bioassay was performed in a 96-well plate, and absorbance was measured at 405 nm.

Real-time PCR

Total RNA was isolated from the liver and kidney tissues using the PureLink RNA Mini Kit (Thermo Fisher Scientific, Waltham, MA, USA). Reverse transcription was performed using the PrimeScript RT Reagent Kit (Takara Bio, Shiga, Japan) according to the manufacturer's protocol. Real-time PCR was conducted using the TB Green Premix Ex Taq II kit (Takara Bio, Shiga, Japan) and the Mx3000 QPCR System (Agilent Technologies, Santa Clara, CA, USA).

Mass analysis of sphingolipids

Total lipids in the liver tissues were extracted with chloroform/methanol (2:1 v/v; 3 mL) at 48 °C for 2 hours. The glycerolipids were saponified by adding 10 M NaOH in water (0.12 mL), and the mixture was incubated at 37 °C for 2 hours. Chloroform (1 mL) and water (1 mL) were added, and the two phases were separated. The hydrophobic phase was collected and analyzed by LC-MS/MS using a TripleTOF 5600 System (AB SCIEX, Foster City, CA) equipped with an electrospray ionization probe and interfaced with a Prominence UFLC system (Shimadzu, Kyoto, Japan), according to a slightly modified reported method.20 The extracted lipids (5 μL) were injected into a column (InertSustain NH_2; particle size, 5 μm; diameter, 2.1 mm; length, 100 mm; GL Science, Tokyo, Japan). Mobile phases A and B consisted of acetonitrile/methanol/formic acid (95:5:0.2, v/v) containing 5 mM ammonium formate and methanol/formic acid (100:0.2, v/v) containing 5 mM ammonium formate, respectively. The lipids were eluted at a rate of 0.13 mL/min through a 45 min solvent gradient: 0–5 minutes, 0% B; 5–10 minutes, from 0% to 20% B; 10–12 minutes, hold at 20% B; 12–15 minutes,

from 20% to 50% B; 15–22 minutes, hold at 50% B; 22–27 minutes, from 50% to 80% B; 27–30 minutes, hold; and 30-45 minutes, from 80% to 0% B. LC-MS/MS analysis was conducted in positive ion mode. C17-Cer and C17-SM were chosen as internal standards. The analytes, d18:2- or d18:2d5-bound Cer and SM, were identified from their retention times and the characteristic product ions (m/z = 264.27 for Cer and m/z = 184.09 for SM). Data acquisition and analysis were performed using the AnalystTF 1.7.1 software (AB SCIEX, Warrington, Cheshire, UK). A bicinchoninic acid assay (Nacalai Tesque) was used to quantify cellular protein.

Statistical analysis

Data are expressed as mean ± standard deviation (SD). Statistical comparisons were made using Student's t-test, Tukey's honestly significant difference (HSD) or one-way analysis of variance, as appropriate, with post hoc analysis performed using GraphPad Prism software (version 9) (GraphPad Software Inc., La Jolla, CA, USA). Statistical significance was set at $p < 0.05$.

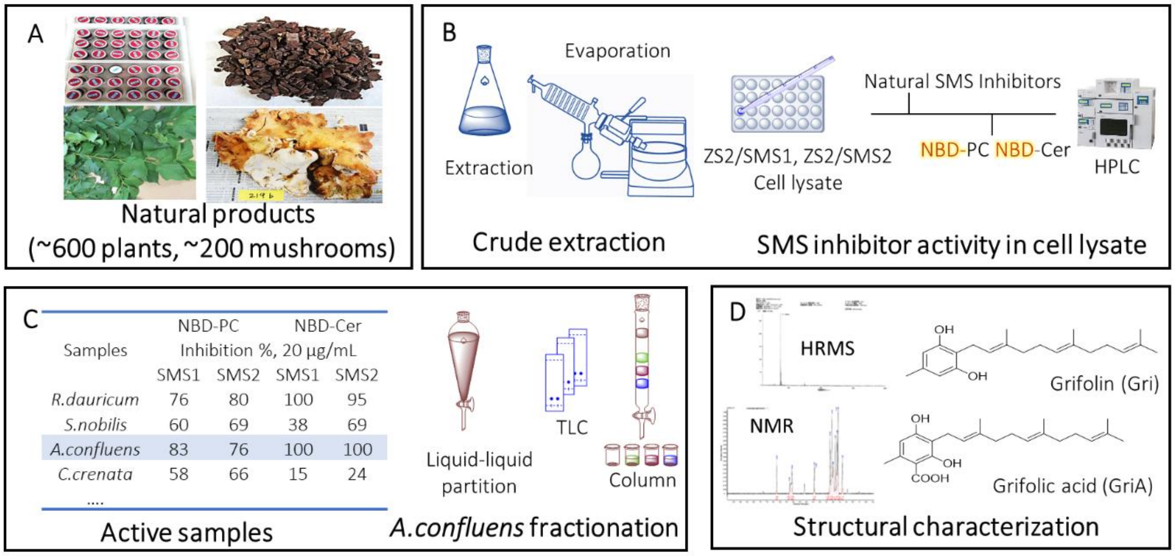

SI Figure 1. Schematic representation of inhibitor of SMS bioassay-guided isolation of natural products. (A) Library of natural products. (B) Crude extraction of natural products. (C) Active samples of SMS inhibition. (D) Structural characterization by HRMS and NMR.

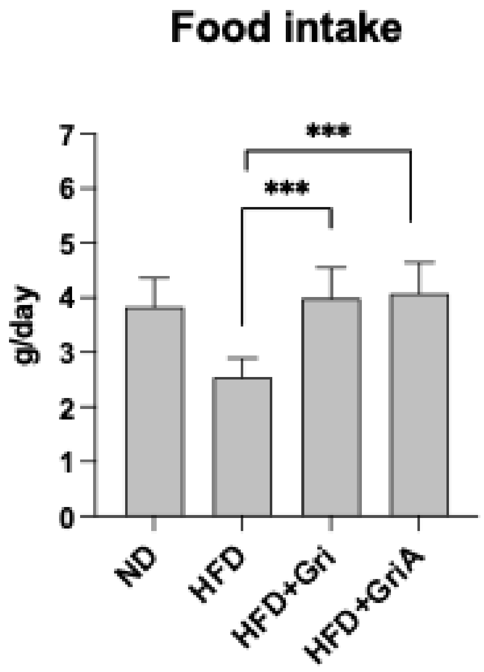

SI Figure 2. In vivo results of grifolin and grifolic acid on food intake

Control: ND, normal chow diet and HFD, High fat diet; Test group: HFD + Gri, HFD treated with 0.1% of grifolin; HFD + GA, HFD treated with o.1% of grifolic acid. Data are presented as mean ± standard error of the mean (SEM); *n* = 6 mice per group. Statistical analysis was done by using t-test: (***) $P < 0.001$.

Compounds	***IC50 µM***			
	SMS 1		***SMS2***	
	Path 1	***Path 2***	***Path 1***	***Path 2***
Grifolin	15.2	0.6	18.3	3
Grifolic acid	3	3	4	2.5
Ginkglic acid (22)	1.5	1.5	-	-
DCA 1 (23)	7	4	-	-
DCA 4 (23)	17	10	-	-
DCA 7 (23)	4	5	-	-
DCA 8 (23)	1.5	1.5	-	-
DCA 9 (23)	2	2	-	-

Malabaricone A (21)	4	4	-	-
Malabaricone B (21)	3.5	2.5	-	-
Malabaricone C (21)	3	1.5	-	-
Malabaricone E (21)	6	4.5	-	-

SI Table 1. Comparison of IC_{50} values (µM) of grifolin, grifolic acid, and previously reported natural SMS inhibitors against SMS1 and SMS2.

Enzyme activity was measured using NBD-ceramide (5 µM) and NBD-phosphatidylcholine (5 µM) as substrates. Path 1 = NBD-PC → NBD-DAG; Path 2 = NBD-Cer → NBD-SM. Previously reported values are cited as: malabaricones (21), ginkgolic acid (22), and daurichromenic acids (23).

A

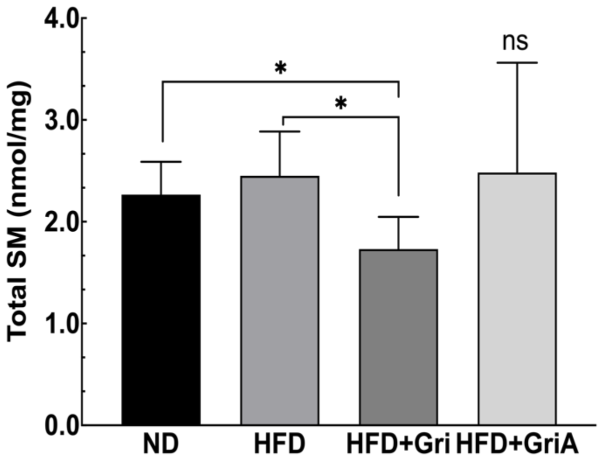

B

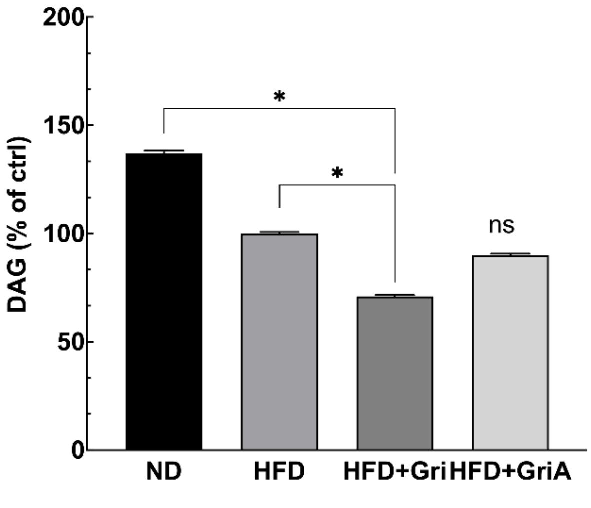

C

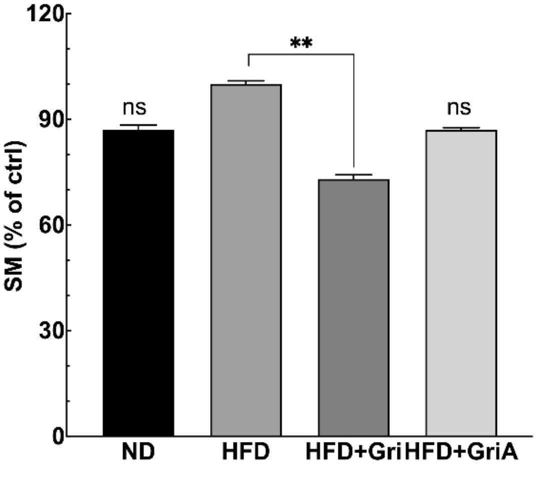

SI Figure 3. SMS inhibitor activity in the liver tissues of mice treated with grifolin and grifolic acid. (A) Total SM levels in the liver tissues. (B) DAG levels synthesized in the liver from PC via SMS. (C) SM levels synthesized in the liver from Cer via SMS. HFD, high-fat diet; Test group: HFD + Gri, HFD supplemented with 0.1% grifolin; HFD + GA, HFD supplemented with 0.1% grifolic acid. Data are presented as mean ± standard error of the mean (SEM); $n = 6$ mice per group. Statistical analysis was performed using one-way ANOVA: (*) $P < 0.05$, (**) $P < 0.01$.

Sample No.	*Scientific name*	*Source Type*
1	*Lyophyllum decastes*	Mushroom
2	*Amanita hemibapha*	Mushroom
3	*Hygrophorus russula*	Mushroom
4	*Cortinarius crocolitus*	Mushroom
5	*Suillus grevillei*	Mushroom
6	*Laccaria amethystea*	Mushroom
7	*Macrolepiota procera*	Mushroom
8	*Amanita pantherina*	Mushroom
9	*Mycoleptodonoides aitchisonii*	Mushroom
10	*Cortinarius vibratilis*	Mushroom
11	*Collybia butyracea*	Mushroom
12	*Clitocybe clavipes*	Mushroom
13	*Lyophyllum connatum*	Mushroom
14	*Laccaria laccata*	Mushroom
15	*Amanita muscaria*	Mushroom
16	*Tricholoma saponaceum*	Mushroom
17	*Tricholoma sejunctum*	Mushroom
18	*Tricholoma robustum*	Mushroom
19	*Cortinarius triumphans*	Mushroom
20	*Boletopsis leucomelas*	Mushroom
21	*Leccinum versipelle*	Mushroom
22	*Polyporellus badius*	Mushroom
23	*Polyporellus brumalis*	Mushroom
24	*Leucoagaricus naucinus*	Mushroom
25	*Armillariella mellea*	Mushroom
26	*Amanita Imazekii*	Mushroom
27	*Pleurotus ostreatus*	Mushroom
28	*Cortinarius purpurascens*	Mushroom
29	*Sarcodon scabrosus*	Mushroom
30	*Piptoporus betulinus*	Mushroom
31	*Tyromyces sambuceus*	Mushroom
32	*Daedalea dickinsii*	Mushroom
33	*Heterobasidion insularis*	Mushroom
34	*Fomes fomentarius*	Mushroom
35	*Phellinus igniarius*	Mushroom
36	*Fomitopsis pinicola*	Mushroom
37	*Elfvingia applanata*	Mushroom
38	*Bondazewia Montana*	Mushroom
39	*Onnia scaura*	Mushroom
40	*Leucopaxillus giganteus*	Mushroom
41	*Naematoloma sublateritium*	Mushroom
42	*Pholiota terrestris*	Mushroom
43	*Lycoperdon perlatum*	Mushroom
44	*Pholiota flammans*	Mushroom
45	*Agaricus abruptibulbus*	Mushroom
46	*Leccinum scabrum*	Mushroom
47	*Phaeolepiota aurea*	Mushroom
48	*Pholiota aurivella*	Mushroom
49	*Pleurotus pulmonarius*	Mushroom
50	*Tricholoma ustale*	Mushroom
51	*Suillus granulatus*	Mushroom
52	*Clitocybe nebularis*	Mushroom
53	*Lepista nuda*	Mushroom
54	*Cortinarius aureobrunneus*	Mushroom
55	*Pholiota nameko*	Mushroom
56	*Tricholoma albobrunneum*	Mushroom
57	*Russula delica var. glaucophylla*	Mushroom
58	*Boletinus cavipes*	Mushroom
59	*Suillus americanus*	Mushroom
60	*Hygrophorus calophyllus*	Mushroom
61	*Hygrophorus chrysodon*	Mushroom
62	*Amanita rubescens*	Mushroom
63	*Tricholoma flavovirens*	Mushroom
64	*Pholiota squarrosa*	Mushroom
65	*Lactarius torminosus*	Mushroom
66	*Lactarius flavidulus*	Mushroom
67	*Lactarius laeticolorus*	Mushroom
68	*Boletus edulis*	Mushroom
69	*Hygrophorus camarophyllus*	Mushroom
70	*Russula sanguinea*	Mushroom
71	*Clitocybe robusta*	Mushroom
72	*Sarcodon leucopus*	Mushroom
73	*Sarcodon aspratus*	Mushroom
74	*Ramaria Formosa*	Mushroom
75	*Lactarius akahatsu*	Mushroom
76	*Russula nigricans*	Mushroom

77	*Naematoloma fasciculare*	Mushroom
78	*Grifola frondosa*	Mushroom
79	*Aleuria aurantia*	Mushroom
80	*Lactarius piperatus*	Mushroom
81	*Cortinarius trivialis*	Mushroom
82	*Lyophyllum fumosum*	Mushroom
83	*Tylopilus fumosipes*	Mushroom
84	*Tricholoma album*	Mushroom
85	*Lactarius necator*	Mushroom
86	*Lactarius scrobiculatus*	Mushroom
87	*Naematoloma gracile*	Mushroom
88	*Lepista irina*	Mushroom
89	*Tricholoma virgatum*	Mushroom
90	*Russula emetica*	Mushroom
91	*Helvella crispa*	Mushroom
92	*Flammulina velutipes*	Mushroom
93	*Hydnellum caeruleum*	Mushroom
94	*Hygrophorus lucorum*	Mushroom
95	*Coriolus versicolor*	Mushroom
96	*Panellus serotinus*	Mushroom
97	*Pholiota spumosa*	Mushroom
98	*Neobulgaria pura*	Mushroom
99	*Tyromyces spumens*	Mushroom
100	*Fuscoporia obliqua*	Mushroom
101	*Schizophyllum commune*	Mushroom
102	*Polyporus alveolaris*	Mushroom
103	*Morchella conica*	Mushroom
104	*Agaricus campestris*	Mushroom
105	*Oudemansiella mucida*	Mushroom
106	*Agrocybe praecox*	Mushroom
107	*Stropharia rugosoannulata*	Mushroom
108	*Psathyrella candolliana*	Mushroom
109	*Polyporus squamosus*	Mushroom
110	*Pleurotus cornucopiae var. citrinopileatus*	Mushroom
111	*Lentinellus ursinus*	Mushroom
112	*Marasmius maximus*	Mushroom
113	*Pholiota albocrenulata*	Mushroom
114	*Agrocybe farinacea*	Mushroom
115	*Auricularia auricula*	Mushroom
116	*Lactarius chrysorrheus*	Mushroom
117	*Tricholomopsis rutilans*	Mushroom
118	*Boletus fraternus*	Mushroom
119	*Boletus subvelutipes*	Mushroom
120	*Russula cyanoxantha*	Mushroom
121	*Phylloporus bellus*	Mushroom
122	*Russula senecis*	Mushroom
123	*Russula foetens*	Mushroom
124	*Tylopilus vinosobrunneus*	Mushroom
125	*Boletus aereus*	Mushroom
126	*Lactarius volemus*	Mushroom
127	*Strobilomyces cofusus*	Mushroom
128	*Tylopilus neofelleus*	Mushroom
129	*Sparassis crispa*	Mushroom
130	*Boletus venenatus*	Mushroom
131	*Collybia acervata*	Mushroom
132	*Pisolithus tinctorius*	Mushroom
133	*Bulgaria inquinans*	Mushroom
134	*Albatrellus caeruleoporus*	Mushroom
135	*Pseudohydnum gelatinosum*	Mushroom
136	*Laetiporus sulphureus var. miniatus*	Mushroom
137	*Pholiota squarrosoides*	Mushroom
138	*Rhodophyllus sinuatus*	Mushroom
139	*Rhodophyllus rhodopolius*	Mushroom
140	*Lactarius uvidus*	Mushroom
141	*Hydnellum concrescens*	Mushroom
142	*Amanita citrina var. citrina*	Mushroom
143	*Descolea flavoannulata*	Mushroom
144	*Phallus impudicus*	Mushroom
145	*Oligoporus tephroleucus*	Mushroom
146	*Clitocybe inornata*	Mushroom
147	*Inocybe fastigiata*	Mushroom
148	*Thelephora palmate*	Mushroom
149	*Calvatia craniiformis*	Mushroom
150	*Lentinellus cochleatus*	Mushroom
151	*Cantharellus cibarius*	Mushroom
152	*Mycena pura*	Mushroom
153	*Amanita subjunquillea*	Mushroom
154	*Russula mariae*	Mushroom
155	*Albatrellus dispansus*	Mushroom
156	*Clitocybe lignatilis*	Mushroom
157	*Stropharia aeruginosa*	Mushroom
158	*Inocybe umbratica*	Mushroom
159	*Paxillus involutus*	Mushroom
160	*Gyrodon lividus*	Mushroom
161	*Coprinus comatus*	Mushroom
162	*Lepista sordida*	Mushroom
163	*Dermocybe cinnamomea*	Mushroom
164	*Collybia maculate*	Mushroom
165	*Hygrophorus eburneus*	Mushroom
166	*Cystoderma amianthinum*	Mushroom
167	*Suillus laricinus*	Mushroom
168	*Psathyrella velutina*	Mushroom
169	*Oudemansiella brunneomaginata*	Mushroom
170	*Gymnopilus liquiritiae*	Mushroom
171	*Leccinum holopus*	Mushroom
172	*Hygrophorus leucophaeus*	Mushroom
173	*Cortinarius allutus*	Mushroom
174	*Rhodophyllus abortivus*	Mushroom
175	*Pholiota lubrica*	Mushroom
176	*Ramaria formosa*	Mushroom
177	*Ramaria flava*	Mushroom
178	*Cortinarius azureus*	Mushroom
179	*Cortinarius xanthophyllus*	Mushroom
180	*Cortinarius duracinus*	Mushroom

181	*Amanita ibotengutake*	Mushroom
182	*Pholiota lenta*	Mushroom
183	*Naematoloma capnoides*	Mushroom
184	*Geastrum triplex*	Mushroom
185	*Lampteromyces japonicus*	Mushroom
186	*Spathularia flavida*	Mushroom
187	*Boletus reticulatus*	Mushroom
188	*Hygrophorus pudorinus*	Mushroom
189	*Laccaria laccata*	Mushroom
190	*Clitocybe gibba*	Mushroom
191	*Camarophyllus virgineus*	Mushroom
192	*Lyophyllum shimeji*	Mushroom
193	*Psathyrella multissima*	Mushroom
194	*Lactarius hysginus*	Mushroom
195	*Clitocybula esculenta*	Mushroom
196	*Pholiota adiposa*	Mushroom
197	*Pseudoclitocybe cyathiformis*	Mushroom
198	*Cortinarius purpurascens*	Mushroom
199	*Tricholoma portentosum*	Mushroom
200	*Armillaria nigrescens Kawam.*	Mushroom
201	*Agaricus subperonatus*	Mushroom
202	*Peziza vesiculosa*	Mushroom
203	*Pluteus atricapillus*	Mushroom
204	*Hypsizigus marmoreus*	Mushroom
205	*Camarophyllus pratensis*	Mushroom
206	*Pleurocybella porrigens*	Mushroom
207	*Ganoderma lucidum KARST.*	Mushroom
208	*Elfvingia applanata KARST.*	Mushroom
209	*Polyporus umbellatus Fries*	Mushroom
210	*Poria cocos Wolf*	Mushroom
211	*Cordyceps sinensis*	Mushroom
212	*Lentinus edodes*	Mushroom
1	*Geranium thunbergii*	Plant
2	*Taxus cuspidata Sieb. et Zucc.*	Plant
3	*Acanthopanax senticosus*	Plant
4	*Aronia melanocarpa*	Plant
5	*Gynostemma pentaphyllum*	Plant
6	*Allium schoenoprasum var. foliosum*	Plant
7	*Convallaria keiskei*	Plant
8	*Celastrus orbiculatus*	Plant
9	*Parthenocissus quinquefolia*	Plant
10	*Hydrangea macrophylla*	Plant
11	*Zelkova serrata*	Plant
12	*Actinidia chinensis*	Plant
13	*Gleditsia japonica*	Plant
14	*Lonicera morrowii (= Caprifolium morrowii)*	Plant
15	*Paulownia tomentosa*	Plant
16	*Cercidiphyllum japonicum*	Plant
17	*Hibiscus syriacus*	Plant
18	*Pueraria lobata*	Plant

19	*Disporum smilacinum*	Plant
20	*Euonymus alatus*	Plant
21	*Sorbus commixta*	Plant
22	*Malus halliana*	Plant
23	*Rhododendron schlippenbachii*	Plant
24	*Enkianthus campanulatus*	Plant
25	*Rhododendron brachycarpum*	Plant
26	*Daphniphyllum macropodum var. humile*	Plant
27	*Chrysanthemum parthenium*	Plant
28	*Prunella vulgaris var. lilacina*	Plant
29	*Plantago lanceolata*	Plant
30	*Arctium lappa*	Plant
31	*Pteridium aquilinum var. latiusculum*	Plant
32	*Vicia cracca*	Plant
33	*Melilotus suaveolens*	Plant
34	*Melilotus alba*	Plant
35	*Malva moschata*	Plant
36	*Glehnia littoralis*	Plant
37	*Gentiana lutea*	Plant
38	*Cnidium officinale*	Plant
39	*Lavandula vera*	Plant
40	*Borago officinalis*	Plant
41	*Silybum marianum (= Carduus maranus)*	Plant
42	*Zanthoxylum piperitum*	Plant
43	*Leonurus sibiricus*	Plant
44	*Perilla frutescens*	Plant
45	*Salvia guaranitica*	Plant
46	*Physalis angulata*	Plant
47	*Carex kobomugi*	Plant
48	*Cynanchum caudatum*	Plant
49	*Codonopsis lanceolata*	Plant
50	*Codonopsis ussuriensis*	Plant
51	*Angelica dahurica*	Plant
52	*Portulaca oleracea*	Plant
53	*Isatis tinctoria var. yezoensis*	Plant
54	*Anaphalis margaritacea var. angustior*	Plant
55	*Allium victorialis var. platyphyllum*	Plant
56	*Cardiocrinum cordatum var. glehnii*	Plant
57	*Smilacina japonica*	Plant
58	*Disporum sessile*	Plant
59	*Polygonatum odoratum var. maximowiczii*	Plant
60	*Polygonatum humile*	Plant
61	*Hosta rectifolia*	Plant
62	*Symphytum officinale*	Plant
63	*Hypericum erectum*	Plant
64	*Convallaria majalis*	Plant
65	*Geranium sibiricum var. glabrius*	Plant

66	*Magnolia kobus var. borealis*	Plant
67	*Magnolia acuminata*	Plant
68	*Platycladus orientalis 'Semperaurea'*	Plant
69	*Catalpa ovata*	Plant
70	*Rosa rugosa*	Plant
71	*Wisteria floribunda*	Plant
72	*Strelitzia reginae*	Plant
73	*Cinnamomum zeylanicum*	Plant
74	*Euphorbia pulcherrima*	Plant
75	*Jacaranda filicifolia*	Plant
76	*Daucus carota*	Plant
77	*Botrychium robustum*	Plant
78	*Rumex obtusifolius*	Plant
79	*Trifolium pratense*	Plant
80	*Metaplexis japonica*	Plant
81	*Solanum nigrum*	Plant
82	*Calystegia japonica*	Plant
83	*Lactuca scariola*	Plant
84	*Erigeron canadensis*	Plant
85	*Conium maculatum*	Plant
86	*Phragmites communis*	Plant
87	*Cicuta virosa*	Plant
88	*Clematis terniflora (= C. paniculata)*	Plant
89	*Polygonum arenastrum*	Plant
90	*Sonchus oleraceus*	Plant
91	*Ginkgo biloba*	Plant
92	*Phellodendron amurense*	Plant
93	*Morus bombycis*	Plant
94	*Robinia pseudo-acacia*	Plant
95	*Aesculus turbinata*	Plant
96	*Vitis coignetiae*	Plant
97	*Helianthus tuberosus*	Plant
98	*Kalopanax pictus*	Plant
99	*Juglans regia var. orientis*	Plant
100	*Thalictrum minus var. hypoleucum*	Plant
101	*Platanus occidentalis*	Plant
102	*Sambucus sieboldiana var. miquelii*	Plant
103	*Chenopodium album var. centrorubrum*	Plant
104	*Cornus controversa*	Plant
105	*Betula platyphylla*	Plant
106	*Cirsium vulgare*	Plant
107	*Commelina communis*	Plant
108	*Solidago gigantea var. leiophylla*	Plant
109	*Hypochoeris radicata*	Plant
110	*Trifolium repens*	Plant
111	*Taraxacum officinale*	Plant
112	*Plantago asiatica*	Plant
113	*Aralia cordata*	Plant
114	*Rorippa sylvestris*	Plant
115	*Potentilla cryptotaeniae*	Plant
116	*Persicaria lapathifolia*	Plant
117	*Raphanus raphanistrum*	Plant
118	*Linaria vulgaris*	Plant
119	*Erigeron annuus*	Plant
120	*Malva neglecta*	Plant
121	*Aster glehnii*	Plant
122	*Lamium barbatum*	Plant
123	*Oenothera biennis*	Plant
124	*Circaea mollis*	Plant
125	*Solanum japonense*	Plant
126	*Acer negundo*	Plant
127	*Polygonum dumetorum*	Plant
128	*Polygonum sachalinense*	Plant
129	*Ampelopsis brevipedunculata*	Plant
130	*Chaenomeles speciosa*	Plant
131	*Petasites japonicus var. giganteus*	Plant
132	*Pilea mongolica*	Plant
133	*Polygonum thunbergii*	Plant
134	*Aster ageratoides form. yezoensis*	Plant
135	*Impatiens noli-tangere*	Plant
136	*Equisetum hyemale*	Plant
137	*Urtica platyphylla*	Plant
138	*Polygonum longisetum*	Plant
139	*Rudbeckia laciniata*	Plant
140	*Verbascum thapsus*	Plant
141	*Euonymus oxyphyllus*	Plant
142	*Lespedeza bicolor*	Plant
143	*Euonymus sieboldianus*	Plant
144	*Malus sieboldii*	Plant
145	*Fraxinus mandshurica var. japonica*	Plant
146	*Alnus japonica*	Plant
147	*Acer mono*	Plant
148	*Trillium kamtschaticum*	Plant
149	*Pachysandra terminalis*	Plant
150	*Geum japonicum*	Plant
151	*Agrimonia pilosa*	Plant
152	*Smilax riparia var. ussuriensis*	Plant
153	*Cardamine leucantha*	Plant
154	*Cacalia hastata var. orientalis*	Plant
155	*Adenocaulon himalaicum*	Plant
156	*Filipendula kamtschatica Maxim.*	Plant
157	*Torilis japonica*	Plant
158	*Aegopodium podagraria*	Plant
159	*Cryptotaenia japonica*	Plant
160	*Sanicula chinensis*	Plant
161	*Phytolacca esculenta*	Plant
162	*Hydrangea petiolaris*	Plant
163	*Sasa senanensis*	Plant
164	*Asperula odorata*	Plant
165	*Lithospermum erythrorhizon*	Plant
166	*Lythrum salicaria*	Plant
167	*Atropa belladonna*	Plant

168	*Phytolacca americana*	Plant
169	*Datura innoxia (= D. meteloides)*	Plant
170	*Scutellaria baikalensis*	Plant
171	*Clematis montana*	Plant
172	*Acanthopanax sieboldianus*	Plant
173	*Coptis japonica*	Plant
174	*Paeonia lactiflora*	Plant
175	*Polygonatum falcatum*	Plant
176	*Echinops ritro*	Plant
177	*Lindera umbellata var. membranacea*	Plant
178	*Zingiber mioga*	Plant
179	*Rosa multiflora*	Plant
180	*Cornus officinalis*	Plant
181	*Cayratia japonica*	Plant
182	*Humulus scandens (= H. japonicus)*	Plant
183	*Polygonum perfoliatum L.*	Plant
184	*Acer saccharum*	Plant
185	*Quercus rubra Linn.*	Plant
186	*Populus sieboldii*	Plant
187	*Castanea crenata*	Plant
188	*Juglans ailanthifolia*	Plant
189	*Ailanthus altissima*	Plant
190	*Ligustrum tschonoskii*	Plant
191	*Dioscorea batatas*	Plant
192	*Chelidonium majus var. asiaticum*	Plant
193	*Stachys japonica villosa*	Plant
194	*Achillea millefolium*	Plant
195	*Epipactis papillosa*	Plant
196	*Duchesnea chrysantha*	Plant
197	*Solidago altissima*	Plant
198	*Senecio vulgaris*	Plant
199	*Ilex crenata var. paludosa*	Plant
200	*Metasequoia glyptostroboides*	Plant
201	*Aesculus glabra*	Plant
202	*Tilia japonica*	Plant
203	*Humulus lupulus*	Plant
204	*Silene dioica*	Plant
205	*Papaver orientale*	Plant
206	*Amaranthus retroflexus*	Plant
207	*Eupatorium rugosum*	Plant
208	*Polygonum filiforme*	Plant
209	*Polygonum nepalense*	Plant
210	*Lilium medeoloides*	Plant
211	*Setaria glauca*	Plant
212	*Sonchus brachyotus (= S. arvensis var. ulginosus)*	Plant
213	*Mentha arvensis var. piperascens*	Plant
214	*Cotoneaster salicifolius*	Plant
215	*Forsythia suspensa*	Plant
216	*Kerria japonica*	Plant
217	*Veronica arvensis*	Plant
218	*Oxalis stricta*	Plant
219	*Bidens tripartita*	Plant
220	*Xanthium strumarium*	Plant
221	*Polygonum cuspidatum*	Plant
222	*Helianthus annuus*	Plant
223	*Rudbeckia hirta var. pulcherrima*	Plant
224	*Cirsium kamtschaticum*	Plant
225	*Aethusa cynapium*	Plant
226	*Albizia julibrissin*	Plant
227	*Coix lacryma-jobi var. mayuen*	Plant
228	*Coffea arabica*	Plant
229	*Hydrangea macrophylla var. thunbergii*	Plant
230	*Corchorus olitorius*	Plant
231	*Angelica keiskei*	Plant
232	*Asarum canadense*	Plant
233	*Liriope platyphylla*	Plant
234	*Ulmus japonica*	Plant
235	*Acer japonicum*	Plant
236	*Berberis amurensis var. japonica*	Plant
237	*Geranium pyrenaicum*	Plant
238	*Aconitum japonicum*	Plant
239	*Artemisia princeps (= A. vulgaris var. indica)*	Plant
240	*Maianthemum dilatatum*	Plant
241	*Amphicarpaea edgeworthii var. japonica*	Plant
242	*Equisetum arvense*	Plant
243	*Sisymbrium officinale*	Plant
244	*Anthemis nobilis*	Plant
245	*Angelica ursina*	Plant
246	*Clematis apiifolia*	Plant
247	*Parthenocissus tricuspidata*	Plant
248	*Clerodendron trichotomum*	Plant
249	*Crassula portulacea*	Plant
250	*Prunus padus*	Plant
251	*Sorbus alnifolia*	Plant
252	*Ostrya japonica*	Plant
253	*Spiraea thunbergii*	Plant
254	*Malus baccata var. mandshurica*	Plant
255	*Lupinus luteus*	Plant
256	*Thladiantha dubia*	Plant
257	*Elsholtzia ciliata*	Plant
258	*Avena sativa*	Plant
259	*Zea mays*	Plant
260	*Oxalis corniculata*	Plant
261	*Asparagus officinalis*	Plant
262	*Ranunculus silerifolius*	Plant
263	*Quercus mongolica var. grosseserrata*	Plant
264	*Acer rubrum*	Plant
265	*Vaccinium ovalifolium*	Plant

266	*Vaccinium hirtum*	Plant
267	*Prunus ssiori*	Plant
268	*Fagus crenata Blume*	Plant
269	*Larix leptolepis*	Plant
270	*Acer palmatum (var. matsumurae)*	Plant
271	*Populus euroamericana*	Plant
272	*Salix bakko*	Plant
273	*Rubus mesogaeus*	Plant
274	*Pinus rigida*	Plant
275	*Pinus koraiensis*	Plant
276	*Pinus strobus*	Plant
277	*Pinus banksiana*	Plant
278	*Picea pungens*	Plant
279	*Pinus thunbergii*	Plant
280	*Saponaria officinalis*	Plant
281	*Spergula arvensis*	Plant
282	*Anemone hupehensis var. japonica*	Plant
283	*Typha latifolia*	Plant
284	*Lilium lancifolium*	Plant
285	*Chenopodium album*	Plant
286	*Digitaria adscendens*	Plant
287	*Medicago sativa*	Plant
288	*Lespedeza cuneata*	Plant
289	*Stachys lanata*	Plant
290	*Capsella bursa-pastoris*	Plant
291	*Ulmus laciniata*	Plant
292	*Prunus avium.*	Plant
293	*Rhus javanica*	Plant
294	*Glycine max*	Plant
295	*Impatiens balfourii*	Plant
296	*Houttuynia cordata*	Plant
297	*Quercus dentata*	Plant
298	*Calendula officinalis*	Plant
299	*Magnolia obovata*	Plant
300	*Calystegia soldanella*	Plant
301	*Thuja occidentalis*	Plant
302	*Spiraea cantoniensis*	Plant
303	*Callicarpa japonica*	Plant
304	*Pyrus pyrifolia var. culta*	Plant
305	*Viscum album var. coloratum*	Plant
306	*Artemisia schmidtiana (= A. sericea var. schmidtiana)*	Plant
307	*Origanum vulgare*	Plant
308	*Datura stramonium*	Plant
309	*Nepeta cataria*	Plant
310	*Verbascum blattaria*	Plant
311	*Cacalia auriculata var. kamtschatica*	Plant
312	*Rubus spp.*	Plant
313	*Saxifraga stolonifera*	Plant
314	*Melissa officinalis*	Plant
315	*Rosa Setigera*	Plant
316	*Trapa japonica*	Plant
317	*Rubus spp.*	Plant
318	*Stellaria media*	Plant
319	*Cochlearia armoracia (= Armoracia rusticana .)*	Plant
320	*Salvia officinalis*	Plant
321	*Solidago virgaurea subsp.. asiatica*	Plant
322	*Artemisia (japonica subsp.) littoricola*	Plant
323	*Aster novi-belgii*	Plant
324	*Ixeris repens*	Plant
325	*Trifolium campestre*	Plant
326	*Elymus mollis*	Plant
327	*Actinidia polygama*	Plant
328	*Rumex acetosa*	Plant
329	*Arabis (stelleri var.) japonica*	Plant
330	*Lathyrus japonicus*	Plant
331	*Styrax obassia*	Plant
332	*Solidago virga-aurea var. leiocarpa*	Plant
333	*Setaria faberi (= S. autumnalis ; S. macrocarpa)*	Plant
334	*Rubus parvifolius*	Plant
335	*Dianthus superbus var. longicalycinus*	Plant
336	*Bidens tripartita*	Plant
337	*Epilobium pyrricholophum*	Plant
338	*Elaeagnus umbellata*	Plant
339	*Hydrangea paniculata*	Plant
340	*Populus alba*	Plant
341	*Pinus montana*	Plant
342	*Eriobotrya japonica*	Plant
343	*Cymbopogon citratus*	Plant
344	*Sterculia nobilis*	Plant
345	*Cocculus laurifolius*	Plant
346	*Ficus elastica*	Plant
347	*Monstera deliciosa*	Plant
348	*Epipremnum aureum*	Plant
349	*Oenanthe javanica*	Plant
350	*Nasturtium officinale*	Plant
351	*Laportea bulbifera*	Plant
352	*Chloranthus japonicus*	Plant
353	*Paris tetraphylla*	Plant
354	*Phlox drummondii*	Plant
355	*Eupatorium chinense var. sachalinense*	Plant
356	*Spuriopimpinella calycina*	Plant
357	*Leibnitzia anandria*	Plant
358	*Tripterospermum japonicum*	Plant
359	*Pyrola renifolia*	Plant
360	*Pyrola incarnata*	Plant
361	*Rubus phoenicolasius*	Plant
362	*Vaccinium japonicum (= Hugeria japonica)*	Plant
363	*Aralia elata*	Plant

364	*Lactuca (raddeana var.) elata*	Plant
365	*Cosmos bipinnatus*	Plant
366	*Alnus hirsuta*	Plant
367	*Viburnum wrightii*	Plant
368	*Tilia maximowicziana*	Plant
369	*Salix integra*	Plant
370	*Actinidia arguta*	Plant
371	*Viburnum furcatum*	Plant
372	*Persicaria sieboldii*	Plant
373	*Berberis thunbergii*	Plant
374	*Maackia amurensis var. buergeri*	Plant
375	*Schizophragma hydrangeoides*	Plant
376	*Sanguisorba tenuifolia var. alba*	Plant
377	*Salix sachalinensis*	Plant
378	*Glaucidium palmatum*	Plant
379	*Boehmeria tricuspis*	Plant
380	*Phryma leptostachya var. asiatica*	Plant
381	*Desmodium fallax var. mandshuricum*	Plant
382	*Impatiens textori*	Plant
383	*Anthriscus sylvestris*	Plant
384	*Angelica genuflexa*	Plant
385	*Angelica edulis*	Plant
386	*Acer mono var. mayrii*	Plant
387	*Daphne kamtschatica var. jezoensis*	Plant
388	*Cimicifuga simplex*	Plant
389	*Aruncus dioicus var. tenuifolius*	Plant
390	*Actaea asiatica*	Plant
391	*Astilbe thunbergii var. congesta*	Plant
392	*Dryopteris crassirhizoma*	Plant
393	*Matteuccia orientalis*	Plant
394	*Osmunda japonica*	Plant
395	*Vinca major*	Plant
396	*Euony fortunei*	Plant
397	*Glechoma hederacea var. grandis*	Plant
398	*Acanthopanax divaricatus*	Plant
399	*Viola kusanoana*	Plant
400	*Fagopyrum esculentum*	Plant
401	*Galinsoga quadriradiata*	Plant
402	*Arisaema serratum (= A. japonica)*	Plant
403	*Chrysanthemum leucanthemum*	Plant
404	*Breea setosa var. subulatum*	Plant
405	*Physalis alkekengi var. franchetii*	Plant
406	*Ilex crenata*	Plant
407	*Perilla frutescens var. acuta forma viridis*	Plant
408	*Hieracium aurantiacum*	Plant
409	*Aster novae-angliae*	Plant
410	*Menyanthes trifoliata*	Plant
411	*Miscanthus sinensis*	Plant
412	*Lycium chinense*	Plant
413	*Akebia quinata*	Plant
414	*Saussurea lappa*	Plant
415	*Ophiopogon japonicus*	Plant
416	*Acer miyabei*	Plant
417	*Lespedeza bicolor var. japonica (= L . bicolor forma actifolia)*	Plant
418	*Equisetum palustre*	Plant
419	*Viburnum sargentii*	Plant
420	*Lotus corniculatus var. corniculatus*	Plant
421	*Polygonum hydropiper*	Plant
422	*Lycopus uniflorus*	Plant
423	*Hydrocotyle ramiflora*	Plant
424	*Actinostemma lobatum*	Plant
425	*Patamogeton distinctus*	Plant
426	*Salix miyabeana*	Plant
427	*Conioselinum kamtschaticum*	Plant
428	*Syringa reticulata*	Plant
429	*Syringa vulgaris*	Plant
430	*Weigela hortensis*	Plant
431	*Lonicera caerulea var. amphyllocalyx*	Plant
432	*Ligustrum obtusifolium*	Plant
433	*Rhamnus japonica var. decipiens (= R. japonica)*	Plant
434	*Sophora flavescens*	Plant
435	*Atractylodes japonica*	Plant
436	*Atractylodes lancea*	Plant
437	*Hemerocallis fulva var. kwanso*	Plant
438	*Solanum sarachoides Sendt.*	Plant
439	*Alangium platanifolium var. trilobum*	Plant
440	*Iris germanica*	Plant
441	*Schizopepon bryoniaefolius*	Plant
442	*Salsola komarovii*	Plant
443	*Antirrhinum majus*	Plant
444	*Mazus miquelii*	Plant
445	*Zinnia elegans; Z. peruviana*	Plant
446	*Artemisia montana*	Plant
447	*Vicia japonica*	Plant
448	*Stellaria aquatica*	Plant
449	*Oplismenus undulatifolius*	Plant
450	*Carpesium abrotanoides*	Plant
451	*Achyranthes fauriei*	Plant
452	*Polystichum tripteron*	Plant
453	*Epimedium grandiflorum subsp. sempervirens . (= E . Sempervirens)*	Plant
454	*Epimedium cremeum*	Plant
455	*Epimedium diphyllum*	Plant
456	*Sium sisarum*	Plant
457	*Macleaya cordata*	Plant
458	*Astragalus membranaceus var. mongholicus (= A. mongholicus)*	Plant

459	*Forsythia viridissima*	Plant
460	*Ephedra distachya Linn*	Plant
461	*Pinellia ternata*	Plant
462	*Allium tuberosum*	Plant
463	*Empetrum nigrum var. japonicum*	Plant
464	*Sparganium stoloniferum*	Plant
465	*Lysimachia vulgaris var. davurica*	Plant
466	*Nigella arvensis*	Plant
467	*Inula japonica (= I. britannica var. japonica)*	Plant
468	*Arachis hypogaea*	Plant
469	*Cynanchum sublanceolatum var. macranthum*	Plant
470	*Weigela coraeensis*	Plant
471	*Gomphocarpus fruticosus*	Plant
472	*Matricaria inodora*	Plant
473	*Erica Linn.*	Plant
474	*Prunus mume*	Plant
475	*Coriaria japonica*	Plant
476	*Cytisus scoparius*	Plant
477	*Rhododendron mucronatum*	Plant
478	*Prunus tomentosa*	Plant
479	*Prunus persica*	Plant
480	*Prunus mume var. bungo*	Plant
481	*Juniperus chinensis var. procumbens*	Plant
482	*Cotoneaster horizontalis*	Plant
483	*Alisma canaliculatum*	Plant
484	*Patrinia scabiosaefolia*	Plant
485	*Astragalus membranaceus*	Plant
486	*Vaccinium smallii*	Plant
487	*Helleborus niger*	Plant
488	*Farfugium japonicum*	Plant
489	*Reynoutria japonica var. compacta f. compacta*	Plant
490	*Polygonum weyrichii*	Plant
491	*Sedum cauticolum*	Plant
492	*Adenophora triphylla var. japonica*	Plant
493	*Lysichiton camtschatcense*	Plant
494	*Carpesium cernuum*	Plant
495	*Polemonium yezoense*	Plant
496	*Asplenium scolopendrium*	Plant
497	*Saxifraga fortunei var. incisolobata*	Plant
498	*Saxifraga japonica*	Plant
499	*Tiarella polyphylla*	Plant
500	*Saxifraga sachalinensis*	Plant
501	*Geranium erianthum*	Plant
502	*Veronica americana*	Plant
503	*Artemisia japonica*	Plant
504	*Agastache rugosa*	Plant
505	*Sedum verticillatum*	Plant
506	*Clinopodium gracile var. sachalinense*	Plant
507	*Bupleurum longiradiatum subsp. sachalinense var. elatius*	Plant
508	*Chimaphila japonica*	Plant
509	*Rumohra standishii*	Plant
510	*Aconitum yesoense*	Plant
511	*Cardamine flexuosa*	Plant
512	*Galium verum var. trachycarpum*	Plant
513	*Chrysosplenium grayanum*	Plant
514	*Woodsia polystichoides*	Plant
515	*Ranunculus repens*	Plant
516	*Achlys japonica*	Plant
517	*Skimmia japonica*	Plant
518	*Euonymus alatus forma striatus*	Plant
519	*Corylus sieboldiana Blume*	Plant
520	*Clematis ochotensis*	Plant
521	*Ilex rugosa*	Plant
522	*Leucothoe grayana var. oblongifolia*	Plant
523	*Ribes japonicum*	Plant
524	*Fraxinus lanuginosa*	Plant
525	*Amorpha fruticosa*	Plant
526	*Acer ukurunduense*	Plant
527	*Actinidia kolomikta*	Plant
528	*Symplocarpus renifolius*	Plant
529	*Veratrum grandiflorum (= V. album var. grandiflorum)*	Plant
530	*Galium trifloriforme F191*	Plant
531	*Eruca sativa*	Plant
532	*Polygonum orientale*	Plant
533	*Solanum melongena cv. [Bei-nasu]*	Plant
534	*Taxus cuspidata var. nana*	Plant
535	*Pinus sylvestris*	Plant
536	*Polygonatum odoratum var. pluriflorum*	Plant
537	*Gaultheria miqueliana*	Plant
538	*Acer palmatum var. sanguineum*	Plant
539	*Rhododendron dauricum*	Plant
540	*Enkianthus perulatus*	Plant
541	*Crataegus pinnatifida*	Plant
542	*Symphoricarpos albus*	Plant
543	*Ribes sanguineum*	Plant
544	*Hamamelis virginiana*	Plant
545	*Narrowleat Meadow Sweet Spiraea*	Plant
546	*Acer diabolicum*	Plant
547	*Larix dahurica var. japonica*	Plant
548	*Acer cissifolium*	Plant
549	*Pterocarya rhoifolia*	Plant
550	*Crataegus arnoldiana*	Plant
551	*Rhamnus purshiana*	Plant
552	*Tussilago farfara*	Plant
553	*Asclepias syriaca*	Plant
554	*Eucommia ulmoides*	Plant
555	*Matricaria chamomilla*	Plant

556	*Ilex serrata (= I. sieboldii ; I. nemotoi)*	Plant
557	*Pieris japonica*	Plant
558	*Phellodendron amurense var. sachalinense*	Plant
559	*Acanthopanax sciadophylloides*	Plant
560	*Rhodotypos scandens*	Plant
561	*Crataegus arduennae (= C. curus-galli)*	Plant
562	*Corylopsis pauciflora*	Plant
563	*Stewartia pseudo-camellia*	Plant
564	*Rosa davurica*	Plant
565	*Crocus sativum*	Plant
566	*Cercis chinensis*	Plant
567	*Zizyphus jujuba var. inermis*	Plant
568	*Physocarpus opulifolius*	Plant
569	*Veronicastrum sibiricum*	Plant
570	*Campsis radicans*	Plant
571	*Apocynum venetum var. basikurumon (Apocynaceae)*	Plant
572	*Ruta graveolens*	Plant
573	*Smilax china*	Plant
574	*Acer nikoense*	Plant
575	*Cornus kousa*	Plant
576	*Salix matsudana tortuosa*	Plant
577	*Rosa heckeliana subsp orientalis*	Plant
578	*Matteuccia struthiopteris*	Plant
579	*Basella rubra*	Plant
580	*Rubus crataegifolius*	Plant
581	*Cydonia oblonga*	Plant
582	*Spiraea faurieana*	Plant
583	*Sorbaria sorbifolia var. stellipila*	Plant
584	*Alnus inokumae*	Plant
585	*Hieracium umbellatum*	Plant
586	*Vicia unijuga*	Plant
587	*Spiraea japonica*	Plant
588	*Staphylea bumalda*	Plant
589	*Crataegus chlorosarca*	Plant
590	*Ribes latifolium*	Plant
591	*Hamamelis japonica var. obtusata*	Plant
592	*Prunus glandulosa var. alboplena*	Plant
593	*Spiraea salicifolia*	Plant
594	*Corylopsis spicata*	Plant
595	*Malus micromalus*	Plant
596	*Stephanandra incisa*	Plant
597	*Chaenomeles japonica*	Plant
598	*Lespedeza cyrtobotrya*	Plant
599	*Prunus japonica*	Plant
600	*Indigofera decora*	Plant
601	*Cynanchum nikoense;*	Plant
602	*Ilex crenata f. bullata*	Plant
603	*Amelanchier asiatica*	Plant
604	*Pharbitis nil*	Plant
605	*Rosmarinus officinalis*	Plant
606	*Magnolia sieboldii*	Plant
607	*Pinus pumila*	Plant
608	*Carpinus laxiflora*	Plant
609	*Philadelphus satsumi*	Plant
610	*Pyrola secunda*	Plant
611	*Crinum asiaticum var. japonicum*	Plant
612	*Hydrangea involucrata*	Plant
613	*Silene armeria*	Plant
614	*Styrax japonica*	Plant
615	*Ulmus pumila*	Plant
616	*Pinus nigra*	Plant
617	*Prunus subhirtella*	Plant
618	*Tsuga diversifolia*	Plant
619	*Acer crataegifolium*	Plant
620	*Liriodendron tulipifera*	Plant
621	*Abies firma*	Plant
622	*Cerasus speciosa*	Plant
623	*Magnolia denudata*	Plant
624	*Cerasus lannesiana;*	Plant
625	*Acer carpinifolium*	Plant
626	*Cynanchum wilfordii*	Plant
627	*Lithospermum officinale*	Plant
628	*Eschscholzia californica*	Plant
629	*Foeniculum vulgare*	Plant
630	*Nepeta cataria; N. minor*	Plant
631	*Lathyrus latifolius*	Plant
632	*Anethum graveolens*	Plant
633	*Lamium purpureum*	Plant
634	*Picrasma quassioides*	Plant
635	*Sedum erythrostictum*	Plant
636	*Ocimum basilicum*	Plant
637	*Calendula officinalis*	Plant
638	*Rheum rhaponticum*	Plant
639	*Cardiospermum halicacabum*	Plant
640	*Sedum kamtschaticum*	Plant
641	*Tanacetum vulgare*	Plant
642	*Lychnis coronaria*	Plant
643	*Celtis jessoensis*	Plant
644	*Hamamelis mollis*	Plant
645	*Diospyros lotus (= L . var. glabra)*	Plant
646	*Magnolia liliflora*	Plant
647	*Prunus triloba*	Plant
648	*Mahonia aquifolium*	Plant
649	*Chamaecyparis pisifera var. fllifera*	Plant
650	*Pterocarya stenoptera*	Plant
651	*Photinia villosa var. laevis*	Plant
652	*Davidia involucrata*	Plant
653	*Diospyros kaki*	Plant
654	*Begonia sp.*	Plant

655	*Lycopodium serratum var. thunbergii*	Plant
656	*Lycopodium clavatum var. nipponicum*	Plant
657	*Aloe arborescens var. natalensis*	Plant
658	*Oxalis articulata*	Plant
659	*Myosotis arvensis*	Plant
660	*Colchicum autumnale*	Plant
661	*Aquilegia flabellata (= A. akitensis)*	Plant
662	*Mirabilis jalapa*	Plant
663	*Papaver rhoeas*	Plant
664	*Tropaeolum majus*	Plant
665	*Dicentra formosa*	Plant
666	*Polygonum cuspidatum forma colorans*	Plant
667	*Rhododendron japonicum Suringer*	Plant
668	*Hypercum androsaemus*	Plant
669	*Persicaria capitata （= Polygonum capitatum）*	Plant
670	*Acanthus mollis*	Plant

SI Table 2. Species list of medicinal plants and mushrooms evaluated for sphingomyelin synthase (SMS) inhibitory activity.

References

S1. Mitsutake S, Zama K, Yokota H, Yoshida T, Tanaka M, Mitsui M, et al. Dynamic modification of sphingomyelin in lipid microdomains controls development of obesity, fatty liver, and type 2 diabetes. *J Biol Chem.* 2011;286:28544–55.

S2. Mitsutake S, Yokose U, Kato M, Matsuoka I, Yoo JM, Kim TJ, et al. The generation and behavioral analysis of ceramide kinase-null mice, indicating a function in cerebellar Purkinje cells. Biochem Biophys *Res Commun.* 2007;363:519–24.
